# Caveolin‐1 Stabilizes SERCA2 to Counteract Acute Kidney Injury via Suppression of Ca^2+^‐Dependent Endoplasmic Reticulum Stress in Distal Tubules

**DOI:** 10.1002/advs.75449

**Published:** 2026-05-08

**Authors:** Yan Zhang, Xin He, Hao Huang, Rong Lu, Xin Lv, Shenglan Li, Sijue Zou, Jiawei Cheng, Yiwei Xiong, Zhenghao Deng, Qiongjing Yuan, Yanyun Xie, Ling Huang, Jiaxi Pu, Shao Liu, Qianbin Li, Jie Meng, Huixiang Yang, Lijian Tao, Zhangzhe Peng

**Affiliations:** ^1^ Department of Nephrology Xiangya Hospital Central South University Changsha China; ^2^ Hunan Key Laboratory of Organ Fibrosis Central South University Changsha China; ^3^ National Clinical Research Center for Geriatric Disorders Xiangya Hospital Central South University Changsha China; ^4^ National Medical Metabolomics International Collaborative Research Center Xiangya Hospital Central South University Changsha China; ^5^ Department of Cell Biology School of Life Sciences Central South University Changsha China; ^6^ Health Management Center Hunan Provincial People's Hospital The First Affiliated Hospital of Hunan Normal University Changsha China; ^7^ Department of Nuclear Medicine Hunan Provincial People's Hospital The First Affiliated Hospital of Hunan Normal University Changsha China; ^8^ Department of Ultrasound Xiangya Hospital Central South University Changsha China; ^9^ Department of Pathology Xiangya Hospital Central South University Changsha China; ^10^ FuRong Laboratory Changsha China; ^11^ Department of Pharmacy Xiangya Hospital Central South University Changsha China; ^12^ Department of Medicinal Chemistry Xiangya School of Pharmaceutical Sciences Central South University Changsha China; ^13^ Department of Pulmonary and Critical Care Medicine Third Xiangya Hospital Central South University Changsha China; ^14^ Department of Gastroenterology Xiangya Hospital Central South University Changsha China

**Keywords:** acute kidney injury, calcium homeostasis, Cav‐1, ER stress, SERCA2

## Abstract

Acute kidney injury (AKI) is a severe clinical condition with high morbidity and mortality. Caveolin‐1 (Cav‐1), a main structural protein of caveolae, orchestrates key cellular processes including endocytosis, lipid transport, and signal transduction by serving as a platform. However, its specific role in AKI remains unclear. Here, we report that Cav‐1 is upregulated in distal tubule epithelial cells (TECs) in both AKI patients and mouse models induced by ischemia/reperfusion injury (IRI) and lipopolysaccharide (LPS). Global and distal TEC‐specific *Cav1* knockout exacerbates IRI and LPS‐induced AKI. RNA‐seq reveals that Cav‐1 deficiency exacerbates intracellular calcium ion (Ca^2+^) homeostasis imbalance and endoplasmic reticulum (ER) stress in injured kidney tissues. Mechanistically, Cav‐1 interacts with sarcoplasmic/endoplasmic reticulum Ca^2+^‐ATPase 2 (SERCA2), a key regulator of intracellular Ca^2+^ homeostasis, through its scaffolding domain, promoting SERCA2 deubiquitination and stability in the ER, thereby maintaining intracellular Ca^2+^ homeostasis and suppressing ER stress in distal TECs. Furthermore, supplementation with a cell‐permeable Cav‐1 scaffolding domain peptide (CSP) or activation of SERCA2 with a small‐molecule agonist CDN1163 alleviates IRI‐ and LPS‐induced AKI, while distal TEC‐specific SERCA2 knockdown abrogates CSP's therapeutic effect. Together, these findings reveal a novel Cav‐1‐mediated pathway and highlight its potential as a therapeutic target for AKI.

## Graphical abstract

In AKI, initial kidney injury upregulates the expression of Cav‐1, which subsequently binds to and stabilizes SERCA2 via its scaffolding domain and through deubiquitination, thereby regulating Ca^2+^ homeostasis and ER stress. Conversely, Cav‐1 deficiency accelerates SERCA2 degradation, triggering Ca^2+^ overload and ER stress, and ultimately exacerbating AKI. The Cav‐1/SERCA2 axis thus serves as a promising therapeutic target for AKI.

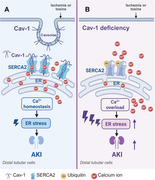

## 1 Introduction

Acute kidney injury (AKI) is a severe clinical syndrome characterized by sudden and often transient loss of renal function. As a life‐threatening condition, AKI affects 20–30% of hospitalized patients and approximately half of intensive care unit (ICU) admissions [1]. Beyond increasing in‐hospital morbidity and mortality, AKI predisposes patients to chronic kidney disease (CKD) and elevates the need for renal replacement therapy [2]. AKI is primarily caused by ischemic kidney injury, often triggered by factors such as multi‐organ failure, transplant surgery, sepsis, or vascular lesions [3]. Currently, clinical management relies on addressing underlying causes, optimizing hemodynamics, and renal replacement therapy when necessary [1]. However, specific treatments remain limited, largely due to an incomplete understanding of the molecular and cellular mechanisms governing tubular injury in AKI. Thus, unraveling these mechanisms is critical for developing effective preventive and therapeutic strategies.

Endoplasmic reticulum (ER) stress emerges as a key pathological process driving tubular cell injury and loss in AKI. Renal tubular injury caused by ischemia or toxins disrupts protein folding homeostasis, leading to misfolded protein accumulation and ER dilation, which triggers ER stress and activates the unfolded protein response (UPR). During AKI, adaptive UPR helps to restore ER homeostasis to promote cell survival and tissue repair. In contrast, uncontrolled and prolonged ER stress becomes cytotoxic, subsequently inducing cell death and exacerbating inflammation [4, 5]. Studies show that sustained ER stress induced by tunicamycin exposure or X‐box binding protein‐1 (XBP1) overexpression results in tubular cell apoptosis and exacerbates tubular necrosis [6, 7], whereas pharmacological inhibition of ER stress or suppression of XBP1 mitigates ischemia/reperfusion injury (IRI) or folic acid‐induced injury [8, 9]. These findings underscore ER stress as a critical pathological factor in AKI, but the underlying mechanisms remain incompletely understood.

Caveolin‐1 (Cav‐1), the core structural component of caveolae, serves as a platform regulating cellular endocytosis, lipid transport, stress responses, and signal transduction. Notably, several studies have shown that Cav‐1 is also distributed in various subcellular compartments, including the Golgi apparatus, the ER, and mitochondria, indicating that Cav‐1 has a non‐caveolae function in the membrane system [10, 11, 12]. Previous studies have demonstrated that Cav‐1 deficiency exacerbates choroidal neovascularization and microglia/macrophage infiltration, and worsens cerebral ischemic injury [13, 14], whereas restoring Cav‐1 expression represses UPR activation and ER stress in vitro and in solid tumors [12]. Additionally, Cav‐1 expression has been reported to increase in injured renal tubules induced by hypoxia or gentamicin [15, 16, 17]. Nevertheless, the specific role of Cav‐1 and its underlying mechanisms in AKI remains undefined.

Here, we demonstrate that Cav‐1 expression is upregulated in distal tubule epithelial cells (TECs) during IRI and lipopolysaccharide (LPS)‐induced injury, as well as in the injured renal tissues from AKI patients. Distal TEC‐specific Cav‐1 deficiency aggravates AKI and ER stress caused by IRI and LPS, whereas administration with a cell‐permeable Cav‐1 scaffolding domain peptide (CSP) improves AKI. The scaffolding domain of Cav‐1 binds to and stabilizes SERCA2 in the ER membrane, a key regulator of intracellular calcium ion (Ca^2+^) homeostasis, thereby alleviating cytoplasmic Ca^2+^ overload and ER stress in TECs. Together, our research supports that dysregulation of the Cav‐1/SERCA2/Ca^2+^ homeostasis/ER functional axis represents a pivotal pathogenic mechanism in AKI, and proposes a potential therapeutic approach for treating AKI.

## 2 Results

### 2.1 Cav‐1 is Significantly Increased in Distal TECs of AKI Patients and Mice

**FIGURE 1:**
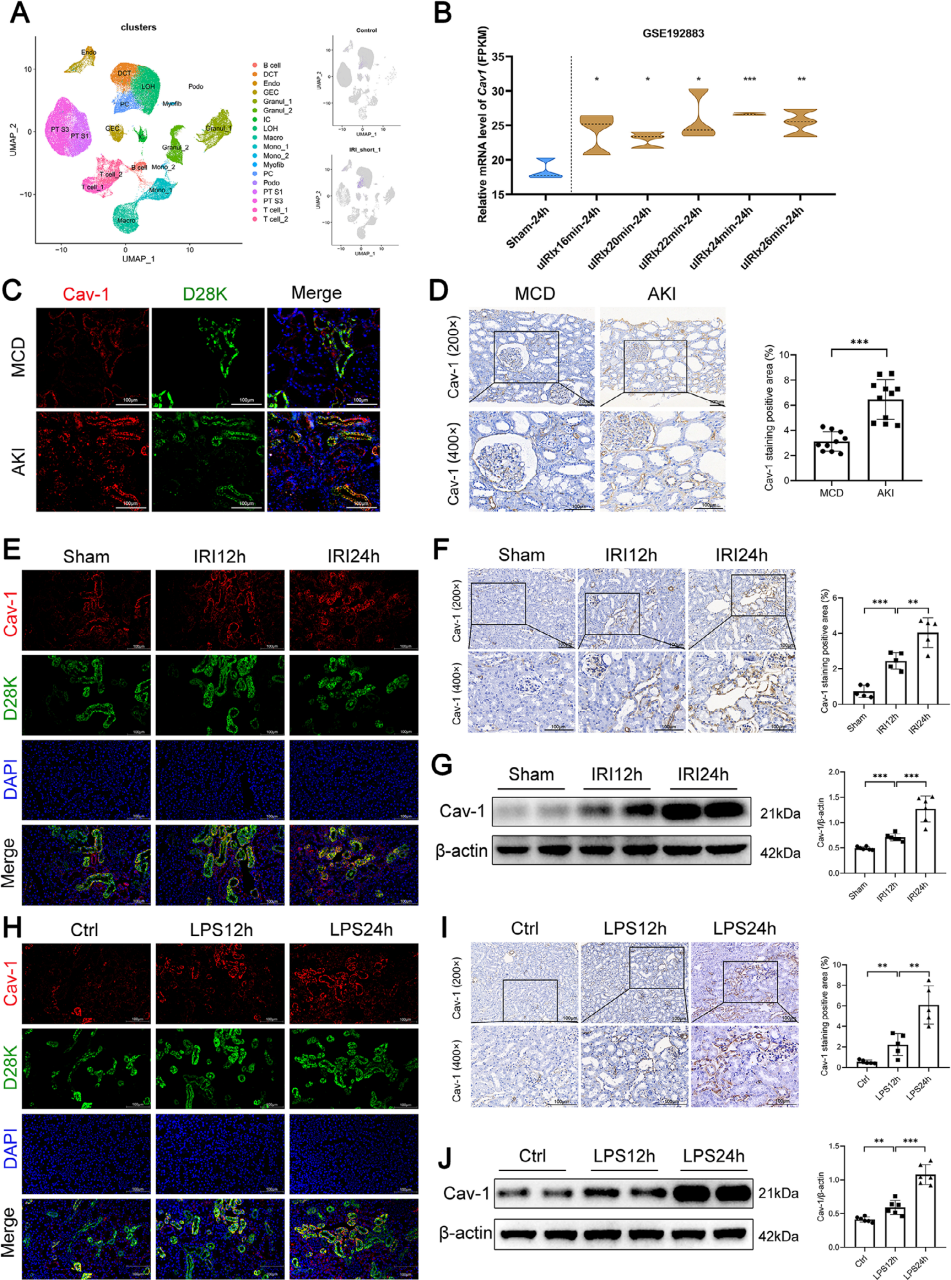
Caveolin‐1 (Cav‐1) is significantly increased in the kidneys of acute kidney injury (AKI) patients and mice models. (A) Composition and distribution of Cav‐1 in human renal single cells from dataset GSE164647. (B) Relative mRNA expression of *Cav1* was significantly upregulated in the mouse kidney sample (dataset GSE192883) after ischemia/reperfusion injury (IRI). (C) Immunofluorescence of Cav‐1 (red) in distal tubules (D28K, green) of the kidney from AKI patients and minimal change disease (MCD) patients. Scale bar = 100 µm. (D) Immunohistochemical (IHC) staining of Cav‐1 in kidney sections from AKI (*n* = 11 samples) and MCD (*n* = 10 samples) patients. Scale bar = 100 µm. (E) Immunofluorescence of Cav‐1 (red) in distal tubules (D28K, green) of the kidney from sham and IRI‐induced AKI mice. Scale bar = 100 µm. (F) IHC staining of Cav‐1 in kidney sections from sham and IRI‐induced AKI mice (*n* = 5 mice per group). Scale bar = 100 µm. (G) Western blot analysis and densitometric quantification of Cav‐1 in kidney tissues from sham and IRI‐induced AKI mice (*n* = 6 mice per group). (H) Immunofluorescence of Cav‐1 (red) in distal tubules (D28K, green) of the kidney from control (Ctrl) and lipopolysaccharide (LPS)‐induced AKI mice. Scale bar = 100 µm. (I) IHC staining of Cav‐1 in kidney sections from Ctrl and LPS‐induced AKI mice (*n* = 5 mice per group). Scale bar = 100 µm. (J) Western blot analysis and densitometric quantification of Cav‐1 in kidney tissues from Ctrl and LPS‐induced AKI mice (*n* = 6 mice per group). Data are expressed as the mean ± SD. ^*^ *p* < 0.05, ^**^ *p* < 0.01, ^***^ *p* < 0.001.

Previous study reported that Cav‐1 is expressed in the glomerulus, distal convoluted tubule, and cortical collecting duct, and renal vessels [18]. Some evidence showed that the expression of Cav‐1 is altered in gentamicin and IRI‐induced AKI [16, 17]. However, little is known regarding its role in AKI. To investigate this, we downloaded the gene expression profiles GSE164647 and GSE192883. Data (GSE164647) from a single‐cell sequencing study of human kidney organoid revealed that *Cav1* expression was higher in the injured group (Figure 1A). Mice mRNA sequencing series GSE192883 showed that *Cav1* also increased significantly in the mice kidney after IRI (Figure 1B). To further validate the upregulation of Cav‐1 in human kidneys with AKI, we first detected Cav‐1 expression in kidney samples from MCD and AKI patients. Additional clinical characteristics are presented in Tables S1 and S2. In MCD patients, renal Cav‐1 was expressed mainly in distal tubular epithelial cells, evident by the colocalization of Cav‐1 and D28k, a marker of distal tubules. Increased Cav‐1 expression was observed in distal tubules of patients with AKI (Figure 1C). Consistently, this phenomenon has also been observed by immunohistochemistry assay (Figure 1D). These results suggest that Cav‐1 upregulation in kidney tissue is a common feature of AKI.

Ischemia and sepsis are two common causes of AKI in clinical settings [3]. Therefore, we established two classical AKI mouse models: ischemic AKI induced by IRI and septic AKI induced by LPS. We evaluated the severity of renal injury by detecting serum creatinine (SCr), blood urea nitrogen (BUN), and renal pathology. As a result, in the IRI‐induced AKI model, the SCr and BUN increased at 12 h of renal reperfusion, and reached even higher at 24 h. HE staining showed renal tubular necrosis, dilation, and more severe damage within 24 h (Figure S1A–D). Importantly, in mice with IRI‐induced AKI, an increase of Cav‐1 in renal distal tubular epithelial cells occurred, and the level of elevation followed the development of AKI (Figure 1E). This result was also confirmed by immunohistochemistry and western blot (Figure 1F, G).

Additionally, we detected the expression of Cav‐1 in the LPS‐induced AKI model. HE staining, SCr, and BUN showed severe tubular injuries in the LPS‐induced AKI group than the normal group (Figure S1E–H). Similar to the findings observed in IRI‐induced AKI mice, the expression of Cav‐1 was also significantly increased in renal distal epithelial cells of LPS‐induced AKI mice (Figure 1H–J). Overall, Cav‐1 expression was significantly elevated in both mouse and human AKI kidneys, strongly suggesting its involvement in the pathogenesis of AKI.

### 2.2 Cav‐1 Deficiency Exacerbates IRI and LPS‐Induced AKI

**FIGURE 2:**
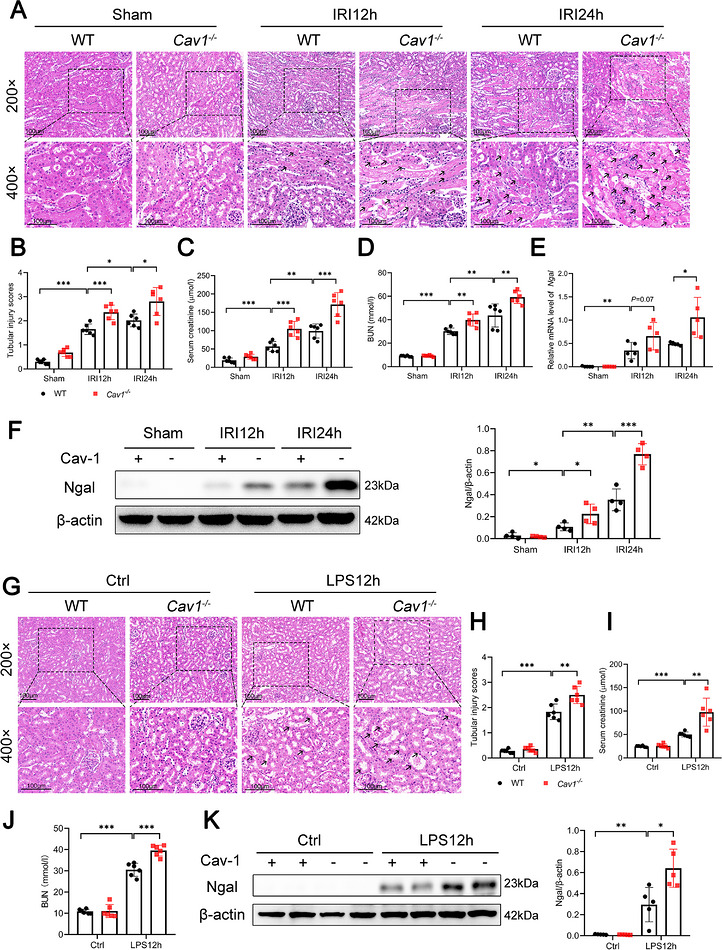
Caveolin‐1 (Cav‐1) deficiency exacerbates IRI and LPS‐induced AKI. (A) Representative images of hematoxylin and eosin (HE) staining of kidney sections from littermate wide type (WT) and Cav‐1 knockout (*Cav1^−/−^*) mice at 12 h and 24 h after IRI (*n* = 6 mice per group). Scale bar = 100 µm. Black arrows indicate injured tubules. (B) Tubular injury scores of kidney tissues (*n* = 6 mice per group). (C), (D) Serum creatinine (SCr) and blood urea nitrogen (BUN) levels of WT and *Cav1^−/−^* mice induced by IRI (*n* = 6 mice per group). (E) Relative mRNA expression of renal neutrophil gelatinase‐associated lipocalin (*Ngal*) in kidney tissues from WT and *Cav1^−/−^* mice induced by IRI (*n* = 5 mice per group). (F) Western blot analysis and densitometric quantification of Ngal in kidney tissues from WT and *Cav1^−/−^* mice induced by IRI (*n* = 4 mice per group). (G) Representative images of HE staining of kidney sections from WT and *Cav1^−/−^* mice induced by LPS (*n* = 6 mice per group). Scale bar = 100 µm. Black arrows indicate injured tubules. (H) Tubular injury scores of kidney tissues (*n* = 6 mice per group). (I), (J) SCr and BUN levels of WT and *Cav1^−/−^* mice induced by LPS (*n* = 6 mice per group). (K) Western blot analysis and densitometric quantification of Ngal in kidney tissues from WT and *Cav1^−/−^* mice induced by LPS (*n* = 5 mice per group). Data are expressed as the mean ± SD. ^*^ *p* < 0.05, ^**^ *p* < 0.01, ^***^ *p* < 0.001.

To investigate the pathological contributions of Cav‐1 to AKI, we examined the impact of Cav‐1 absence on AKI using *Cav1* knockout (*Cav1^−/−^*) and littermate wild type (WT) mice. Western blot revealed the absence of Cav‐1 expression in the kidney of *Cav1^−/−^* mice (Figure S2). We detected the kidney histology and serum renal function of 2‐month‐old *Cav1^−/−^* mice and found that there were no phenotypically abnormalities or renal dysfunction. Subsequently, both *Cav1^−/−^* and WT mice were subjected to IRI and sacrificed 12 and 24 h after reperfusion. Hematoxylin and eosin (HE) staining revealed that *Cav1^−/−^* mice suffered more severe tubular injuries compared with WT models (Figure 2A, B). Serum renal function tests showed that the levels of SCr and BUN were significantly higher in *Cav1^−/−^* mice compared with WT mice (Figure 2C, D). Besides, consistent with the pathological and serological findings, RT‐PCR and Western blot revealed that the mRNA and protein expression of Ngal were significantly increased in the kidneys of *Cav1^−/−^* mice (Figure 2E, F).

Similar results were also observed in *Cav1^−/−^* mice treated with LPS. HE staining and serum renal function tests showed more severe tubular injuries in LPS models of *Cav1^−/−^* mice (Figure 2G–J). In addition, the protein expression of Ngal also confirmed the aggravation of kidney injury in *Cav1^−/−^* mice (Figure 2K). Taken together, these results suggested Cav‐1 plays a protective role in the progression of AKI.

### 2.3 Distal TEC‐Specific Cav‐1 Deletion Exacerbates IRI and LPS‐Induced AKI

**FIGURE 3:**
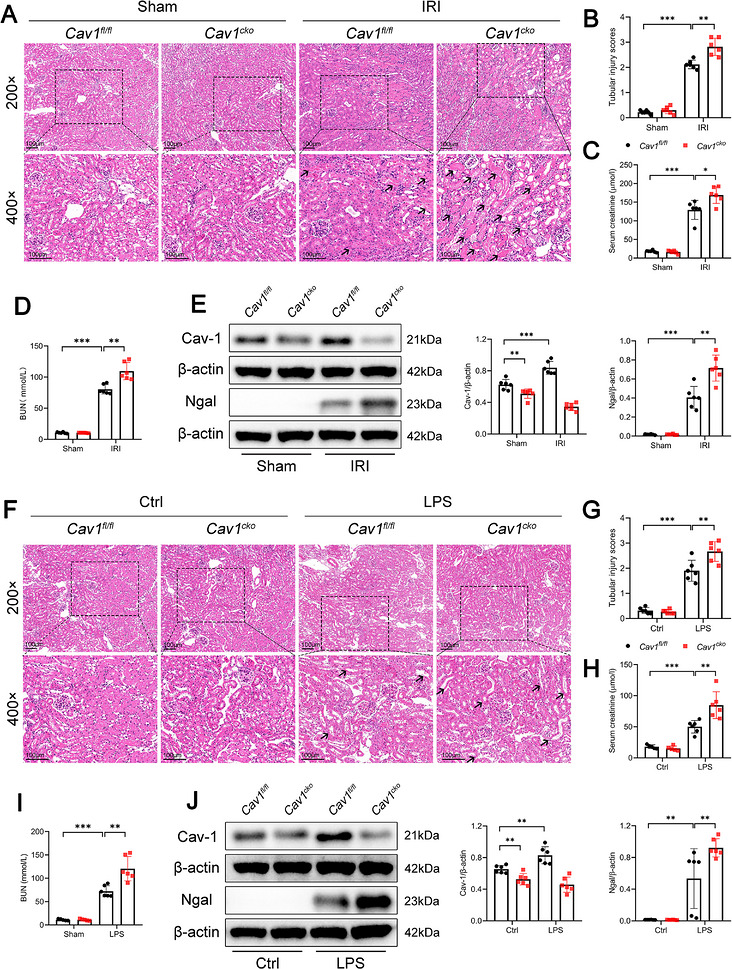
Distal TECs specific deletion of Cav‐1 exacerbates IRI and LPS‐induced AKI. (A) Representative images of hematoxylin and eosin (HE) staining of kidney sections from control (*Cav1^fl/fl^*) and TECs conditional *Cav‐1* knockout (*Cav1^cko^*) mice at 24 h after IRI (*n* = 6 mice per group). Scale bar = 100 µm. Black arrows indicate injured tubules. (B) Tubular injury scores of kidney tissues (*n* = 6 mice per group). (C), (D) Serum creatinine (SCr) and blood urea nitrogen (BUN) levels of *Cav1^fl/fl^* and *Cav1^cko^* mice induced by IRI (*n* = 6 mice per group). (E) Western blot analysis and densitometric quantification of Cav‐1 and Ngal in kidney tissues from *Cav1^fl/fl^* and *Cav1^cko^* mice induced by IRI (*n* = 6 mice per group). (F) Representative images of HE staining of kidney sections from *Cav1^fl/fl^* and *Cav1^cko^* mice induced by LPS (*n* = 6 mice per group). Scale bar = 100 µm. Black arrows indicate injured tubules. (G) Tubular injury scores of kidney tissues (*n* = 6 mice per group). (H), (I) SCr and BUN levels of *Cav1^fl/fl^* and *Cav1^cko^* mice induced by LPS (*n* = 6 mice per group). (J) Western blot analysis and densitometric quantification of Ngal in kidney tissues from *Cav1^fl/fl^* and *Cav1^cko^* mice induced by LPS (*n* = 6 mice per group). Data are expressed as the mean ± SD. ^*^ *p* < 0.05, ^**^ *p* < 0.01, ^***^ *p* < 0.001.

Based on the obvious upregulation of Cav‐1 in distal tubules, we generated distal tubule‐specific *Cav1* knockout mice using CRISPR‐Cas9 technology to better elucidate the role of Cav‐1 in renal tubular epithelial cells. Cdh16‐Cre mice (mice expressing Cre recombinase under the cadherin 16 promoter) were crossed with *Cav1^fl/fl^* mice to generate distal tubule‐specific *Cav1* knockout mice (*Cav1^fl/fl^*‐Cre, also called *Cav1^cko^* mice), which was confirmed by tail genotyping (Figure S3). These *Cav1^cko^* mice did not exhibit significant renal dysfunction under physiological conditions. However, according to the HE staining, Cav‐1 deficiency in distal TECs significantly exacerbated IRI‐induced pathological changes such as degeneration and necrosis of renal tubular epithelial cells (Figure 3A, B). Concurrently, compared with *Cav1^fl/fl^* mice, *Cav1^cko^* mice displayed exacerbated renal dysfunction, with elevated SCr and BUN levels (Figure 3C, D). Western blot showed that the expression of Cav‐1 in the IRI‐induced injured renal cortex was significantly upregulated, whereas the protein expression of *Cav1^cko^* mice was significantly reduced compared to control mice (Figure 3E). Moreover, the role of Cav‐1 deficiency in IRI‐AKI mice was further confirmed by increased expression of Ngal and Kim‐1 (Figure 3E; Figure S4A).

We similarly used the LPS‐induced AKI mouse model to verify the role of Cav‐1 in distal TECs in AKI. Based on HE staining and tubular injury scores, distal tubule‐specific knockout of *Cav1* aggravated LPS‐induced tubular injuries. This was further corroborated by elevated SCr, BUN, and the expression of Ngal and Kim‐1 (Figure 3F–J; Figure S4B).

### 2.4 Cav‐1 Scaffolding Domain Peptide (CSP) Alleviates IRI and LPS‐Induced AKI in Both WT and *Cav1^−/−^* Mice

**FIGURE 4:**
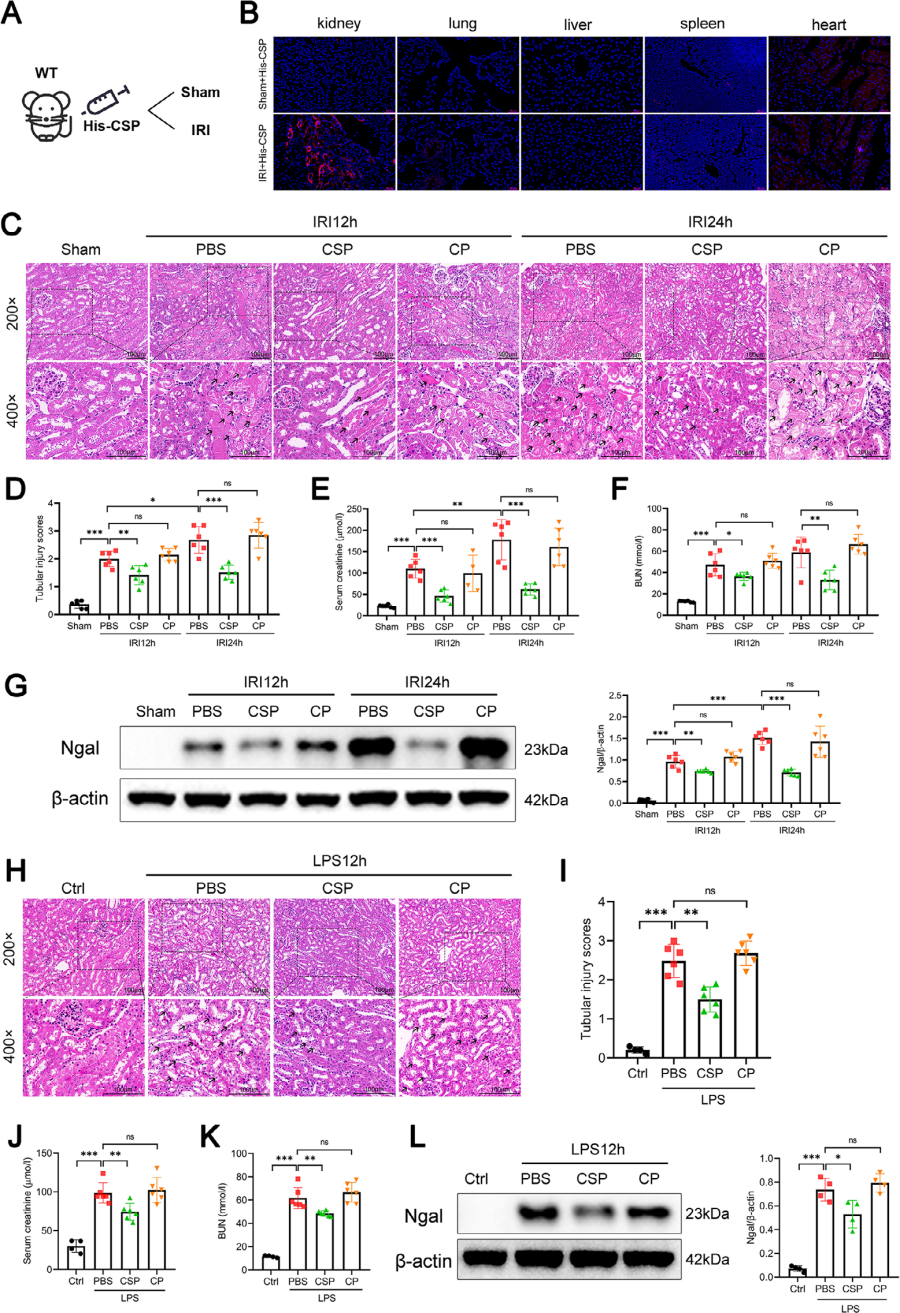
Cav‐1 scaffolding domain peptide (CSP) alleviates IRI and LPS‐induced AKI in *Cav1^−/−^* mice. (A) Scheme of the experimental approach. WT mice subjected to sham or IRI operation were i.p. injected with His‐tagged CSP (1.5 mg/kg) 12 h before surgery, and major organs were collected 12 h after surgery. (B) Representative micrographs of immunofluorescence of His‐CSP (red) in organs. Tissue sections were stained with an anti‐His antibody. (C) *Cav1^−/−^* mice were i.p. injected with CSP or scrambled control peptide (CP) 12 h before IRI surgery and sacrificed 12 or 24 h after reperfusion. Representative images of hematoxylin and eosin (HE) staining of kidney sections from *Cav1^−/−^* mice induced by IRI (*n* = 6 mice per group). Scale bar = 100 µm. Black arrows indicate injured tubules. (D) Tubular injury scores of kidney tissues (*n* = 6 mice per group). (E), (F) Serum creatinine (SCr) and blood urea nitrogen (BUN) levels in different groups of *Cav1^−/−^* mice (*n* = 6 mice per group). (G) Western blot analysis and densitometric quantification of Ngal in kidney tissues from *Cav1^−/−^* mice (*n* = 6 mice per group). (H) *Cav1^−/−^* mice were i.p. injected with CSP or CP 4 h before LPS injection and sacrificed 12 h later. Representative images of HE staining of kidney sections from *Cav1^−/−^* mice induced by LPS (*n* = 6 mice per group). Scale bar = 100 µm. Black arrows indicate injured tubules. (I) Tubular injury scores of kidney tissues (*n* = 6 mice per group). (J), (K) SCr and BUN levels in different groups of *Cav1^−/−^* mice (*n* = 6 mice per group). (L) Western blot analysis and densitometric quantification of Ngal in kidney tissues from *Cav1^−/−^* mice (*n* = 4 mice per group). Data are expressed as the mean ± SD. ^*^ *p* < 0.05, ^**^ *p* < 0.01, ^***^ *p* < 0.001, ns not significant.

CSP is a cell‐permeable peptide derived from Cav‐1 [19]. Previous studies have demonstrated that cell‐permeable CSP can ameliorate vascular inflammation, inhibit vascular leakage, and suppress pathological neovascularization in the ocular choroid by mimicking the function of Cav‐1 [13, 19, 20]. Given that Cav‐1 deficiency exacerbated AKI, we hypothesized that CSP might play a reno‐protective role in AKI. To this end, we first examined the tissue distribution of His‐tagged CSP (His‐CSP) in vivo. His‐CSP was injected i.p. into both sham‐operated mice and IRI‐induced AKI mice (Figure 4A). Major organs were collected 12 h after reperfusion, immunofluorescence staining of tissue sections revealed that His‐CSP was predominantly accumulated in the injured tubular epithelial cells, whereas other major organs including liver, lung, heart, and spleen, as well as the uninjured kidneys of sham‐operated mice, exhibited little accumulation of His‐CSP, suggesting that CSP is preferentially delivered to the injured tubules (Figure 4B).

To verify the effect of CSP in AKI, we constructed AKI models in both *Cav1^−/−^* and WT mice and treated with CSP, respectively. CSP or scrambled control peptide (CP) was administered 12 h before IRI surgery by i.p. injection, and 4 h before LPS injection. HE staining showed fewer tubular injuries in *Cav1^−/−^* mice with CSP treatment (Figure 4C, D), renal function analysis of SCr and BUN also revealed that CSP could significantly ameliorate IRI in *Cav1^−/−^* mice (Figure 4E, F). In addition, the expression of Ngal was significantly decreased after CSP treatment (Figure 4G). We also observed that CSP treatment alleviated tubular damage and improved renal function in LPS‐induced AKI models of *Cav1^−/−^* mice (Figure 4H–L). Similarly, we verified the protective role of CSP in WT mice. Beyond the initial prophylactic administration, which effectively alleviated renal dysfunction in both IRI and LPS‐induced AKI of WT mice (Figure S5), we further investigated the therapeutic potential of CSP. Notably, administration of CSP 1 h after IRI or LPS injection also significantly mitigated tubular damage and restored renal function in WT mice (Figure S6). These results suggest that CSP protects against the onset and progression of AKI caused by various factors and further validate the protective role of Cav‐1 in AKI.

### 2.5 Cav‐1 Regulates Intracellular Calcium Homeostasis and Endoplasmic Reticulum Stress In Vivo

**FIGURE 5:**
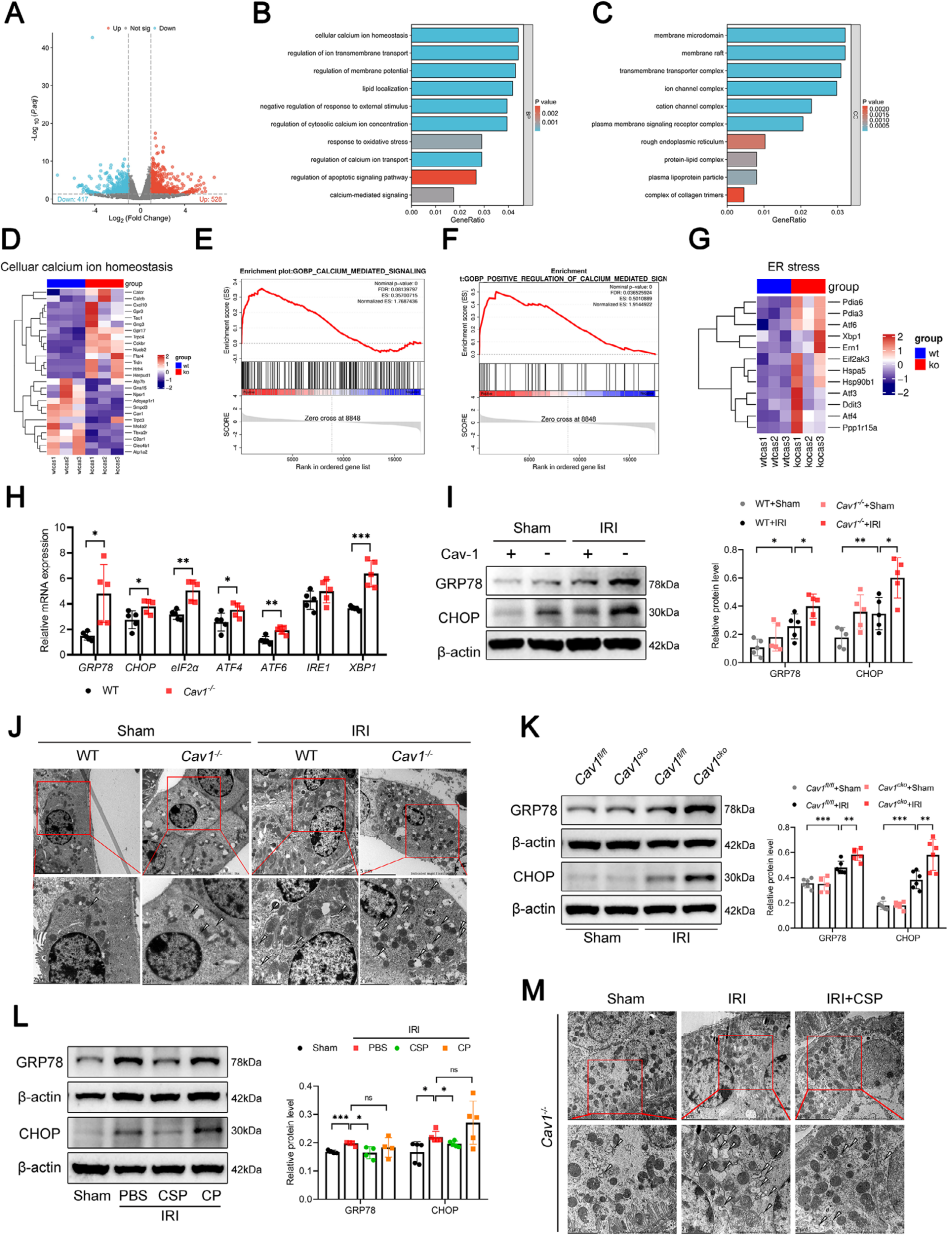
Cav‐1 regulates cellular calcium ion homeostasis and endoplasmic reticulum (ER) stress in vivo. (A) The volcano map visualized the distribution of differential genes (including up‐regulation and down‐regulation) in the kidney tissues of WT and *Cav1^−/−^* mice induced by IRI. (B) GO cluster analysis illustrating the Biological Process (top 10) of WT and *Cav1^−/−^* mice after IRI. (C) GO cluster analysis illustrating the Cellular Component (top 10) of WT and *Cav1^−/−^* mice induced by IRI. (D) The heat map of cellular calcium ion homeostasis pathway from GO enrichment in kidney tissues of WT and *Cav1^−/−^* mice after IRI. (E), (F) GSEA analysis of calcium‐mediated signaling in kidney tissues of WT and *Cav1^−/−^* mice after IRI. (G) The heat map of expression of ER stress‐related genes in kidney tissues of WT and *Cav1^−/−^* mice after IRI. (H) Relative mRNA expression of *GRP78, CHOP, eIF2α, ATF4, ATF6, IRE1, and XBP1* in kidney tissues from WT and *Cav1^−/−^* mice induced by IRI (*n* = 6 mice per group). (I) Western blot analysis and densitometric quantification of GRP78 and CHOP in kidney tissues from WT and *Cav1^−/−^* mice (*n* = 5 mice per group). (J) Typical images of transmission electron microscopy (TEM) of kidney sections from WT and *Cav1^−/−^* mice at 12 h after IRI. At 5000× magnification, scale bar = 5 µm; at 10000× magnification, scale bar = 2 µm. (K) Western blot analysis and densitometric quantification of GRP78 and CHOP in kidney tissues from *Cav1^fl/fl^* and *Cav1^cko^* mice (*n* = 6 mice per group). (L) Western blot analysis and densitometric quantification of GRP78 and CHOP in kidney tissues from sham, CSP, and CP‐treated *Cav1^−/−^* mice (*n* = 4–5 mice per group). (M) Typical images of TEM of kidney sections from *Cav1^−/−^* mice induced by IRI with CSP treatment. At 5000× magnification, scale bar = 5 µm; at 10000× magnification, scale bar = 2 µm. Data are expressed as the mean ± SD. ^*^ *p* < 0.05, ^**^ *p* < 0.01, ^***^ *p* < 0.001, ns not significant.

To investigate the cellular mechanism by which Cav‐1 regulates AKI, we performed RNA sequencing (RNA‐seq) on kidney tissues from IRI‐induced WT and *Cav1^−/−^* mice. A volcano plot was constructed based on the differentially expressed transcripts, which identified 417 downregulated and 528 upregulated transcripts in IRI‐induced *Cav1^−/−^* mice compared with WT mice (Figure 5A). The Gene Ontology (GO) cluster analysis, including the Biological Process (BP) and Cellular Component (CC), revealed that Cav‐1 may be closely associated with cellular Ca^2+^ homeostasis, membrane function, ion channels, and the endoplasmic reticulum (Figure 5B, C). Consistently, mRNA levels of genes involved in the Ca^2+^ homeostasis pathways in GO enrichment analyses exhibited significant alterations (Figure 5D). Gene set enrichment analysis (GSEA) further validated the enrichment of calcium‐mediated signaling pathways (Figure 5E, F). Ca^2+^ homeostasis within the ER serves as a major player in maintaining intracellular calcium signaling [21]. Disruption of ER Ca^2+^ homeostasis leads to unfolded protein accumulation, which is the major driver of ER stress [22, 23]. Accordingly, mRNA expression analysis of ER stress‐related genes, including *Pdia6, Pdia3, ATF6, Xbp1, Ern1, Eif2ak3, and Hspa5*, revealed significant upregulation in *Cav1^−/−^* mice compared with WT mice (Figure 5G).

Consistently, RT‐PCR analysis revealed significant upregulation of mRNA levels of ER stress‐related indicators in IRI‐injured kidney tissues of *Cav1^−/−^* mice compared to the WT mice (Figure 5H). We further investigated the regulatory effect of Cav‐1 on key protein expressions of the ER stress pathway, and observed that Cav‐1 deficiency also increased GRP78 and CHOP protein expression in kidney tissues of AKI mice (Figure 5I). As ER expansion is a morphological hallmark of ER stress caused by the accumulation of misfolded or unfolded proteins [24], we further assessed ultrastructural changes of the tubular epithelial cells of kidneys subjected to IRI injury using transmission electron microscopy (TEM). TEM analysis showed apparent ER dilation in distal TECs of WT mice after IRI‐induced AKI, which was more pronounced in *Cav1^−/−^* mice (Figure 5J). Additionally, a significant reduction in ER‐mitochondria contact sites was observed in the kidneys of *Cav1^−/−^* mice (Figure S7). Furthermore, the upregulation of GRP78 and CHOP in the renal cortex of *Cav1^cko^* mice confirmed the modulatory role of Cav‐1 in distal tubules on ER stress during AKI (Figure 5K). Importantly, supplementation with CSP blunted the increased expression of GRP78 and CHOP and attenuated ER expansion in IRI‐induced kidney tissues of *Cav1^−/−^* mice (Figure 5L, M).

### 2.6 Cav‐1 Regulates Intracellular Calcium Homeostasis and Endoplasmic Reticulum Stress In Vitro

**FIGURE 6:**
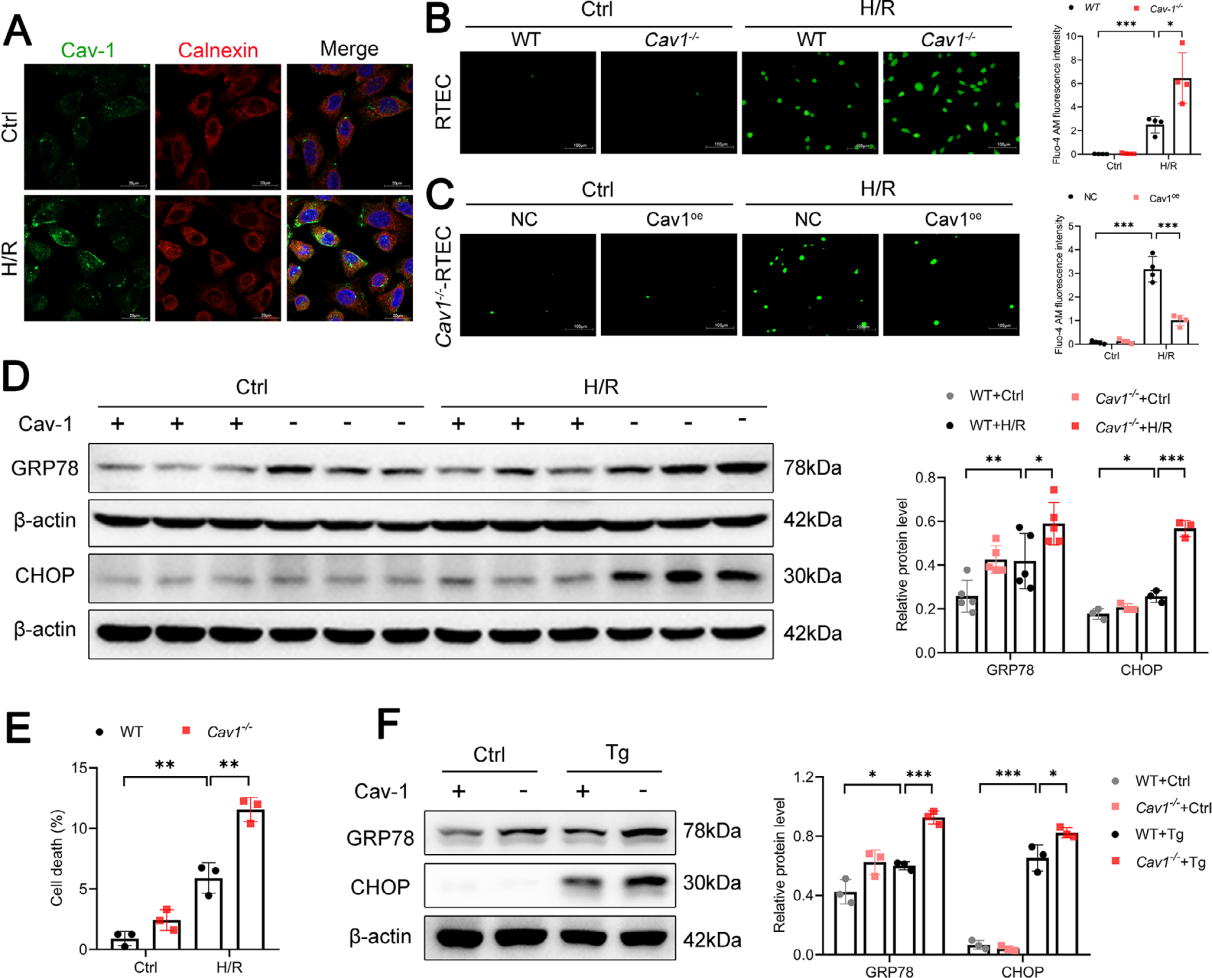
Cav‐1 regulates cellular calcium ion homeostasis and endoplasmic reticulum (ER) stress in vitro. (A) Cav‐1 expression was increased in the ER in H/R induced RTECs isolated from WT mice. Representative fluorescence images of Cav‐1 expression (green) in the ER (Calnexin, red) of primary RTECs with or without 6 h of hypoxia followed by 4 h of reoxygenation. Scale bar = 20 µm. (B) Representative of Fluo‐4 AM fluorescent staining of WT and *Cav1^−/−^* primary RTECs induced by H/R. Scale bar = 100 µm. (C) Representative of Fluo‐4AM fluorescence staining of *Cav1^−/−^* primary RTECs treated with negative control (NC) or Cav‐1 overexpression (*Cav1* ^OE^) after H/R. (D) Western blot analysis and densitometric quantification of GRP78 and CHOP in WT and *Cav1^−/−^* primary RTECs induced by H/R (*n* = 3–5 biologically independent cells). (E) LDH release rate of WT and *Cav1^−/−^* primary RTECs induced by H/R (*n* = 3 biologically independent cells). (F) Western blot analysis and densitometric quantification of GRP78 and CHOP in WT and *Cav1^−/−^* primary RTECs induced by thapsigargin (Tg) for 3 h (*n* = 3 biologically independent cells). Scale bar = 100 µm. Data are expressed as the mean ± SD. ^*^ *p* < 0.05, ^**^ *p* < 0.01, ^***^ *p* < 0.001, ns not significant.

Based on RNA‐seq results from IRI‐induced kidney tissues, the regulation of Cav‐1 on intracellular Ca^2+^ homeostasis and ER stress pathways was further examined in primary renal tubular epithelial cells (RTECs). Laser confocal microscopy demonstrated a marked increase in Cav‐1 expression within ER compartments in RTECs isolated from WT mice after hypoxia/reoxygenation (H/R) injury, which was confirmed by co‐localization with the ER marker calnexin (Figure 6A). Using the Ca^2+^ fluorescence probe Fluo‐4/AM, we observed significantly elevated intracellular Ca^2+^ concentration in H/R‐treated *Cav1^−/−^* primary RTECs compared with WT primary RTECs (Figure 6B). Conversely, Cav‐1 overexpression alleviated Ca^2+^ overload in *Cav1^−/−^* primary RTECs (Figure 6C). Further investigation was conducted to explore the effect of Cav‐1 on ER stress in H/R‐induced RTECs. Western blot analysis revealed that H/R significantly upregulated GRP78 and CHOP in primary RTECs, and Cav‐1 deficiency exacerbated these effects (Figure 6D). And Cav‐1 deficiency exacerbates H/R‐induced primary RTECs cell death, evidenced by increased LDH release (Figure 6E). Moreover, we also treated the primary RTECs with the ER stress inducer thapsigargin (Tg), and the results revealed that Cav‐1 deficiency aggravated Tg‐induced ER stress as well (Figure 6F). Furthermore, supplementation with CSP mitigated H/R‐induced ER stress in *Cav1^−/−^* primary RTECs (Figure S8). Taken together, our results collectively demonstrate that Cav‐1 alleviates the injury of TECs by modulating Ca^2+^ homeostasis and suppressing ER stress.

### 2.7 Cav‐1 Directly Interacts with SERCA2 through the Scaffolding Domain and Regulates Its Protein Stability through Deubiquitination

**FIGURE 7:**
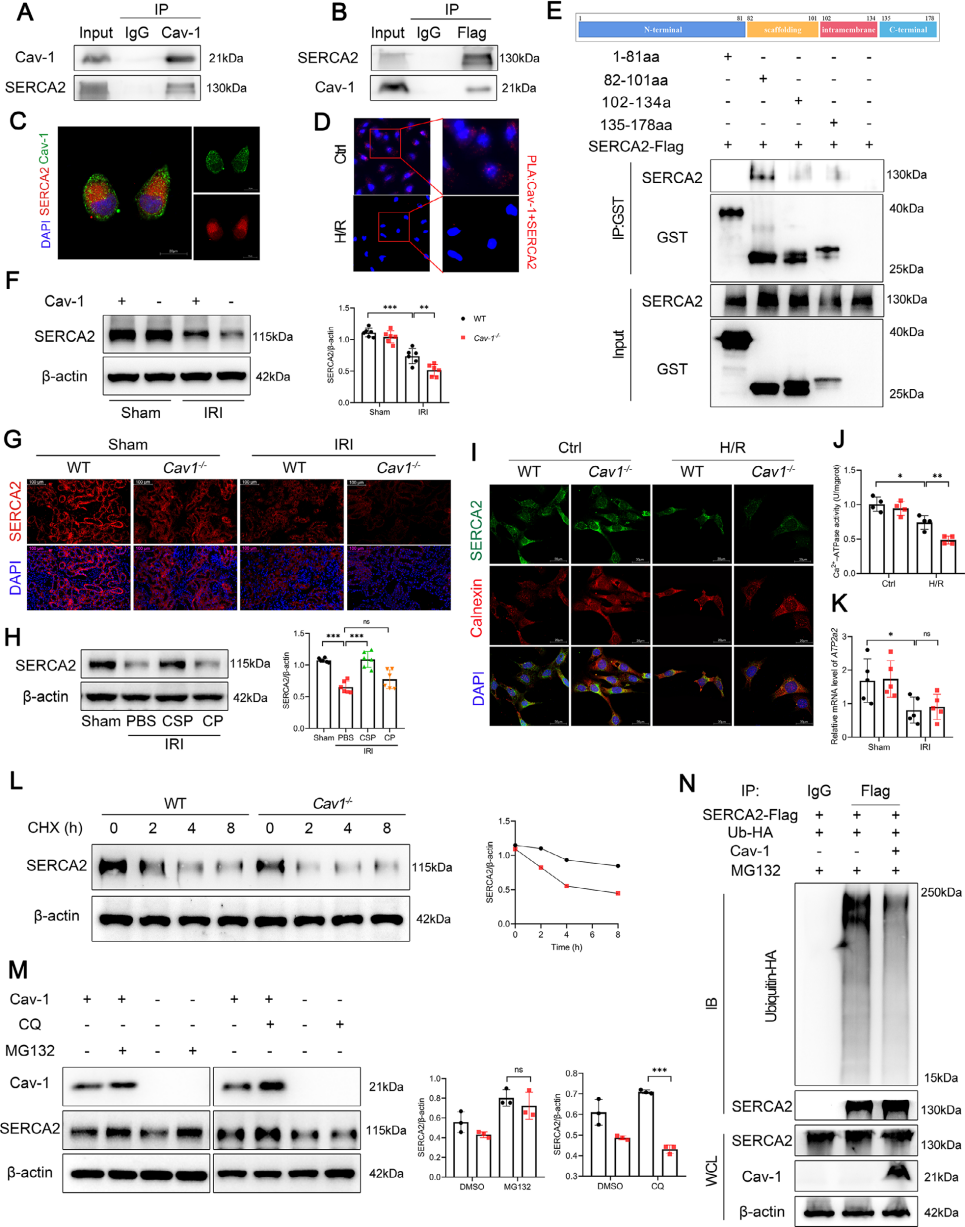
Cav‐1 interacts with SERCA2 through the scaffolding domain and regulates its protein stability through deubiquitination. (A), (B) Co‐immunoprecipitation (Co‐IP) analysis of Cav‐1 with SERCA2 in HEK293T cells co‐transfected with overexpression plasmids of Cav‐1 and Flag‐tagged SERCA2. (C) Confocal analysis of Cav‐1 (green) and SERCA2 (red) in primary RTECs induced by H/R. (D) Representative images of the proximity ligation assay (PLA) demonstrating the endogenous spatial proximity between Cav‐1 and SERCA2 in RTECs. (E) Schematic illustration of the Cav‐1 domain truncated plasmids and Co‐IP analysis of four Cav‐1 truncated domains with SERCA2 in HEK293T cells co‐transfected with overexpression plasmids of GST‐tagged truncated Cav‐1 and Flag‐tagged SERCA2. (F) Western blot analysis and densitometric quantification of SERCA2 in kidney tissues from WT and *Cav1^−/−^* mice subjected to sham operation or 12 h after IRI operation (*n* = 6 mice per group). (G) Immunofluorescence of SERCA2 (red) of kidney from WT and *Cav1^−/−^* mice subjected to sham operation or 12 h after IRI operation. Scale bar = 100 µm. (H) Western blot analysis and densitometric quantification of SERCA2 in kidney tissues from *Cav1^−/−^* mice induced by IRI with Cav‐1 scaffolding domain peptide (CSP) or scrambled control peptide (CP) treatment (*n* = 6 mice per group). (I) Representative fluorescence images of SERCA2 (green) expression in the ER (Calnexin, red) of WT and *Cav1^−/−^* primary RTECs treated with or without H/R. Scale bar = 20 µm. (J) Ca^2+^‐ATPase activity of WT and *Cav1^−/−^* primary RTECs treated with or without H/R (*n* = 4 biologically independent cells). (K) Relative mRNA expression of *ATP2a2* in kidney tissues from WT and *Cav1^−/−^* mice induced by IRI (*n* = 5 mice per group). (L) Cycloheximide (CHX) chase assay for SERCA2 in WT and *Cav1^−/−^* primary RTECs treated with CHX (500 µg mL^−1^) for the indicated time points. (M) Western blot analysis of Cav‐1 and SERCA2 of WT and *Cav1^−/−^* primary RTECs treated with MG132 (10 µm) or CQ (10 µm) for 8 h (*n* = 3 biologically independent cells). (N) Co‐IP assay for the ubiquitination of SERCA2 in HEK293T cells transfected with overexpression plasmids of Cav‐1, Flag‐tagged SERCA2, and HA‐tagged ubiquitin plasmids, and treated with MG132. Data are expressed as the mean ± SD. ^*^ *p* < 0.05, ^**^ *p* < 0.01, ^***^ *p* < 0.001, ns not significant.

After revealing that Cav‐1 deficiency can aggravate ER stress during AKI, we next sought to understand the mechanisms behind. To investigate the specific mechanism by which Cav‐1 regulates Ca^2+^ homeostasis and ER stress, we transfected primary RTECs with a Cav‐1 overexpression plasmid and screened for potential Cav‐1 substrate proteins using co‐immunoprecipitation (Co‐IP) combined with mass spectrometry. Notably, SERCA2 (sarcoplasmic/endoplasmic reticulum Ca^2+^‐ATPase 2), a key transporter regulating sarcoplasmic/endoplasmic reticulum (SR/ER) Ca^2+^ influx [25], emerged as the top candidate and may be a potential target of Cav‐1 (Figure S9). During cardiac contraction, SERCA2 is responsible for trafficking cytosolic free Ca^2+^ back into the SR/ER [25]. However, little is known about the role of SERCA2 in AKI. To confirm the association between Cav‐1 and SERCA2, we co‐transfected Cav‐1 and Flag‐tagged SERCA2 overexpression plasmids into HEK293T cells. Co‐IP showed that Cav‐1 and SERCA2 were strongly associated (Figure 7A, B). Confocal imaging experiments utilizing these probes showed evidence of partial colocalization of Cav‐1 with SERCA2 in primary RTECs induced by H/R (Figure 7C). To further validate the relationship between Cav‐1 and SERCA2, proximity ligation assay (PLA) revealed significantly fewer Cav‐1‐SERCA2 proximity signals in H/R‐induced primary RTECs vs. controls (Figure 7D). Cav‐1 has four domains: N‐terminal (residues 1–81), scaffolding (residues 82–101), intramembrane (residues 102–134), and C‐terminal (residues 135–178) [19]. To further clarify which domain of Cav‐1 binds to SERCA2, we constructed four Cav‐1 truncated plasmids with a GST tag, respectively. Co‐transfection of truncated GST‐tagged Cav‐1 plasmids and Flag‐tagged SERCA2 overexpression plasmids in HEK293T cells revealed that SERCA2 binds to the Cav‐1 scaffolding domain (Figure 7E). Taken together, these results show that Cav‐1 binds to SERCA2 directly via its scaffolding domain.

The interaction between Cav‐1 and SERCA2 raises the question of whether Cav‐1 plays a regulatory role in SERCA2 expression and function. To this end, we investigated the effect of Cav‐1 on SERCA2 in IRI‐induced AKI models. Western blot analysis and immunofluorescence images revealed reduced SERCA2 expression in IRI‐induced AKI, with a more pronounced downregulation observed in *Cav1* ^−/−^ mice (Figure 7F, G). Notably, supplementation of CSP rescued the expression of SERCA2 in the kidney tissues from *Cav1^−/−^* mice (Figure 7H). To further determine the impact of Cav‐1 on SERCA2, we detected SERCA2 expression in primary RTECs induced by H/R. Immunofluorescence imaging confirmed that SERCA2 is localized in the ER, and its protein levels decreased under H/R injury, Cav‐1 deficiency further exacerbated this downregulation (Figure 7I). Given the regulatory effect of Cav‐1 on SERCA2 expression in tubular epithelial cells, we measured Ca^2+^‐ATPase activity (SERCA2 enzyme activity) in primary RTECs. Consistent with protein expression data, the activity of SERCA2 was reduced in H/R‐induced primary RTECs, and this reduction was further exaggerated in *Cav1^−/−^* RTECs (Figure 7J). Importantly, the effect of Cav‐1 on SERCA2 expression and activity was not due to altered transcription of SERCA2 (encoded by *ATP2a2*) (Figure 7K).

Considering that Cav‐1 influences SERCA2 protein levels but not mRNA expression, we hypothesized that Cav‐1 prevents SERCA2 degradation through regulation of post‐translational modifications rather than transcription. Ubiquitination is one of the most common post‐translational modifications, and the expression and activity of SERCA2 have been shown to be regulated by ubiquitination [26, 27]. Therefore, we set out to explore whether Cav‐1 regulates SERCA2 ubiquitination. Cycloheximide (CHX) chase experiments showed that Cav‐1 deficiency shortened the half‐life of SERCA2 protein in primary RTECs, suggesting that Cav‐1 can prevent SERCA2 from proteasomal degradation (Figure 7L). To explore the specific ways in which Cav‐1 deficiency accelerates SERCA2 degradation, we treated primary RTECs with the proteasome inhibitor MG132 and the autophagy inhibitor CQ. And we found that treatment with MG132, but not CQ, rescued SERCA2 protein levels in *Cav1^−/−^* primary RTECs (Figure 7M). Additionally, we co‐transfected Flag‐tagged SERCA2 and HA‐tagged ubiquitin with or without Cav‐1 overexpression plasmids in HEK293T cells, and then treated the cells with MG132. Overexpression of Cav‐1 significantly reduced the binding of SERCA2 to ubiquitin (Figure 7N). These results demonstrate that Cav‐1 regulates calcium homeostasis in kidney injury AKI by stabilizing the SERCA2 protein via the deubiquitination pathway.

### 2.8 The Protective Effect of CSP Against IRI‐Induced AKI is Abrogated when SERCA2 is Knockdown

Analysis of public single‐cell RNA sequencing databases revealed abundant SERCA2 expression in renal tubules (Figure S10). As mentioned before, Cav‐1 mainly increased in the distal tubules of AKI models. Therefore, to elucidate the physiological and pathological relationship between Cav‐1 and SERCA2 in the kidney, we used CRISPR‐Cas9 technology to create a mouse model of conditionally SERCA2 (encoded by *ATP2a2*) knocked out in distal TECs (Cdh16‐Cre). We found that homozygous conditional knockout mice reached full term but died within one week of birth, which is consistent with previous reports of death in mice with mutations of SERCA2 [28]. Therefore, heterozygous conditionally knockdown adult mice were used in this experiment (*ATP2a2^fl/+^‐Cre*). PCR was utilized to confirm conditional *ATP2a2* knockdown using specific primers, indicating successful model construction (Figure S11A–C).

**FIGURE 8:**
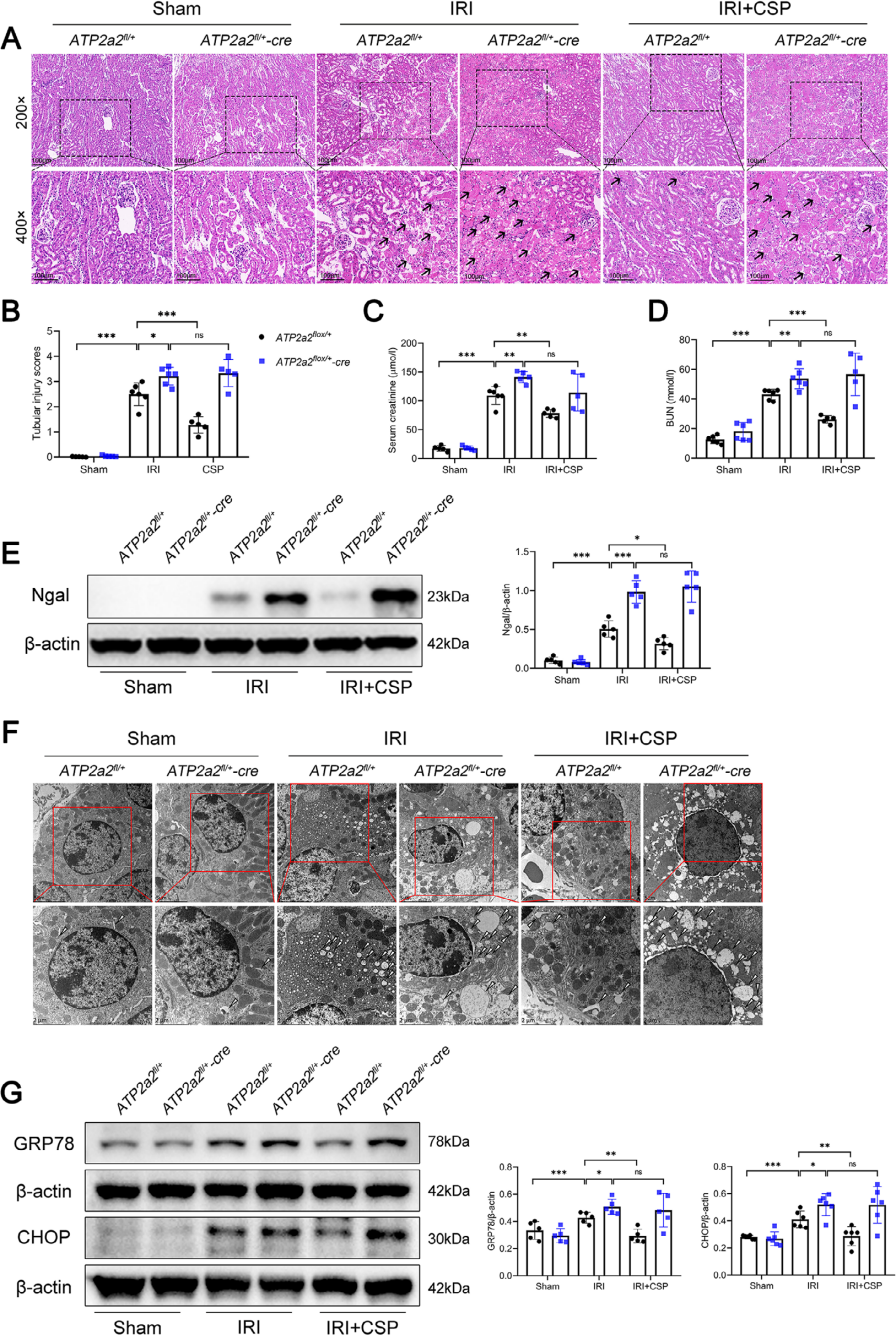
The protective effect of CSP against IRI‐induced AKI is abrogated when SERCA2 is knocked down. SERCA2 distal tubular conditional knock‐down (*ATP2a2^fl/+^‐Cre*) and control (*ATP2a2^fl/+^*) mice were i.p. injected with vehicle or CSP 12 h before IRI and sacrificed 12 h after reperfusion. (A) Representative images of HE staining of kidney sections from *ATP2a2^fl/+^* and *ATP2a2^fl/+^‐Cre* mice (*n* = 5–6 mice per group). Scale bar = 100 µm. Black arrows indicate injured tubules. (B) Tubular injury scores of kidney tissues (*n* = 5–6 mice per group). (C), (D) SCr and BUN levels in different groups of *ATP2a2^fl/+^* and *ATP2a2^fl/+^‐Cre* mice (*n* = 5–6 mice per group). (E) Western blot analysis and densitometric quantification of Ngal in kidney tissues from *ATP2a2^fl/+^* and *ATP2a2^fl/+^‐Cre* mice (*n* = 5 mice per group). (F) Typical images of TEM of kidney sections from *ATP2a2^fl/+^* and *ATP2a2^fl/+^‐Cre* mice. At 5000× magnification, scale bar = 5 µm; at 10000× magnification, scale bar = 2 µm. (G) Western blot analysis and densitometric quantification of GRP78 and CHOP in kidney tissues from *ATP2a2^fl/+^* and *ATP2a2^fl/+^‐Cre* mice. Data are expressed as the mean ± SD. ^*^ *p* < 0.05, ^**^ *p* < 0.01, ^***^ *p* < 0.001, ns not significant.

Then, we constructed an IRI‐induced AKI model using *ATP2a2^fl/+^‐Cre* and control mice (*ATP2a2^fl/+^*), and CSP treatment was provided 12 h before IRI surgery. As presented in Figure 8A, B, compared with IRI control mice, SERCA2 conditional knockdown aggravated the tubular injuries of *ATP2a2^fl/+^‐Cre* mice with IRI‐induced AKI. SCr and BUN levels, expression of Ngal protein were higher in *ATP2a2^fl/+^‐Cre* mice with IRI compared to those in control mice (Figure 8C–E). TEM images revealed greater ER expansion in the distal TECs of *ATP2a2^fl/+^‐Cre* mice induced by IRI compared with control mice (Figure 8F). Western blot analyses also showed that GRP78 and CHOP were significantly elevated in the kidneys of *ATP2a2^fl/+^‐Cre* mice with IRI (Figure 8G). These results suggested that conditional SERCA2 knockdown exacerbates renal injuries and ER stress in mice with IRI‐induced AKI. More importantly, consistent with the results observed in *Cav1^−/−^* and WT mice, i.p. injection of CSP alleviates IRI‐induced AKI in control mice, while the renoprotective effect of CSP was abolished in *ATP2a2^fl/+^‐Cre* mice (Figure 8A–E). Intriguingly, TEM images and the analysis of GRP78 and CHOP showed that the role of CSP in regulating ER stress in AKI was significantly abrogated in *ATP2a2^fl/+^‐Cre* mice compared with control mice (Figure 8F, G). Together, these results suggested that SERCA2 is the key downstream regulator of Cav‐1 in IRI‐induced AKI and ER stress.

### 2.9 SERCA2 Activation and Overexpression Alleviate IRI‐Induced AKI in *Cav1* ^−/−^ Mice

**FIGURE 9:**
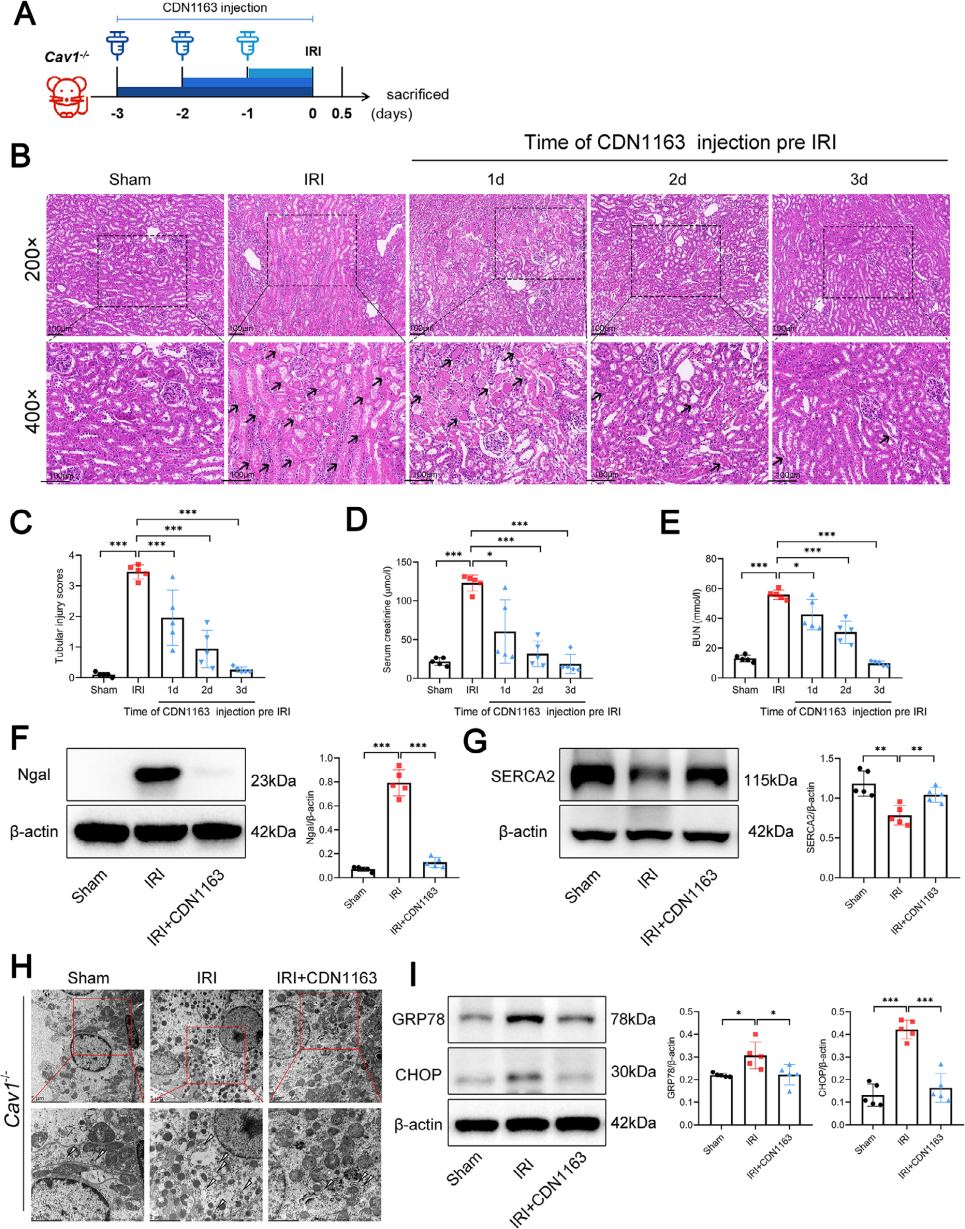
SERCA2 activation and overexpression alleviate IRI‐induced AKI in *Cav1^−/−^* mice. (A) The experimental flowchart for the treatment of SERCA2 activator (CDN1163) in *Cav1^−/−^* mice induced by IRI: *Cav1^−/−^* mice were i.p. injected with CDN1163 every day for 1, 2, or 3 days before IRI surgery and sacrificed 12 h after reperfusion. (B) Representative images of HE staining of kidney sections from *Cav1^−/−^* mice induced by IRI with CDN1163 treatment (*n* = 5 mice per group). Scale bar = 100 µm. Black arrows indicate injured tubules. (C) Tubular injury scores of kidney tissues (*n* = 5 mice per group). (D), (E) SCr and BUN levels in different groups (*n* = 5 mice per group). (F) Western blot analysis and densitometric quantification of Ngal in kidney tissues from different groups (*n* = 5 mice per group). (G) Western blot analysis and densitometric quantification of SERCA2 in kidney tissues from different groups of *Cav1^−/−^* mice (*n* = 5 mice per group). (H) Typical images of TEM of kidney sections from different groups of *Cav1^−/−^* mice. At 5000× magnification, scale bar = 5 µm; at 10000× magnification, scale bar = 2 µm. (I) Western blot analysis and densitometric quantification of GRP78 and CHOP in kidney tissues from different groups of *Cav1^−/−^* mice (*n* = 5 mice per group). Data are expressed as the mean ± SD. ^*^ *p* < 0.05, ^**^ *p* < 0.01, ^***^ *p* < 0.001.

After revealing that SERCA2 is a key regulator for the renoprotective role of Cav‐1 in AKI, we attempted to determine whether activation or overexpression of SERCA2 could serve as a potential therapeutic strategy for AKI treatment. *Cav1^−/−^* mice were i.p. injected with CDN1163, the activator of SERCA2, once daily for 1, 2, or 3 days prior to IRI surgery (Figure 9A). Compared with vehicle‐treated IRI‐induced *Cav1^−/−^* mice, mice treated with CDN1163 showed significantly reduced pathological renal tubular injury, as well as lower SCr and BUN levels. Moreover, longer durations of CDN1163 pretreatment resulted in greater improvements (Figure 9B–E). Meanwhile, we evaluated the therapeutic efficacy of CDN1163 administration, and found that i.p. injection of CDN1163 1 h post‐reperfusion also ameliorated tubular injury and renal dysfunction in *Cav1^−/−^* mice (Figure S12A–E). Western blot showed that CDN1163 treatment decreased Ngal expression and increased SERCA2 expression, indicating SERCA2 upregulation alleviated IRI‐induced AKI in *Cav1^−/−^* mice (Figure 9F, G). Furthermore, TEM images and western blot analysis showed that activation of SERCA2 in *Cav1^−/−^* mice restored ER expansion in distal TECs and reduced the expression of GRP78 and CHOP during AKI to comparable levels of IRI models (Figure 9H, I). In addition, in vitro experiments showed that transfection of the SERCA2 overexpression plasmid into *Cav1^−/−^* primary RTECs significantly improved Ca^2+^ overload in H/R‐induced cellular models (Figure S13). In conclusion, these results further prove that SERCA2 is the key downstream effector of Cav‐1 in regulating calcium homeostasis and ER stress in AKI, and that targeting SERCA2 is a viable therapeutic strategy for AKI treatment.

## 3 Discussion

**FIGURE 10:**
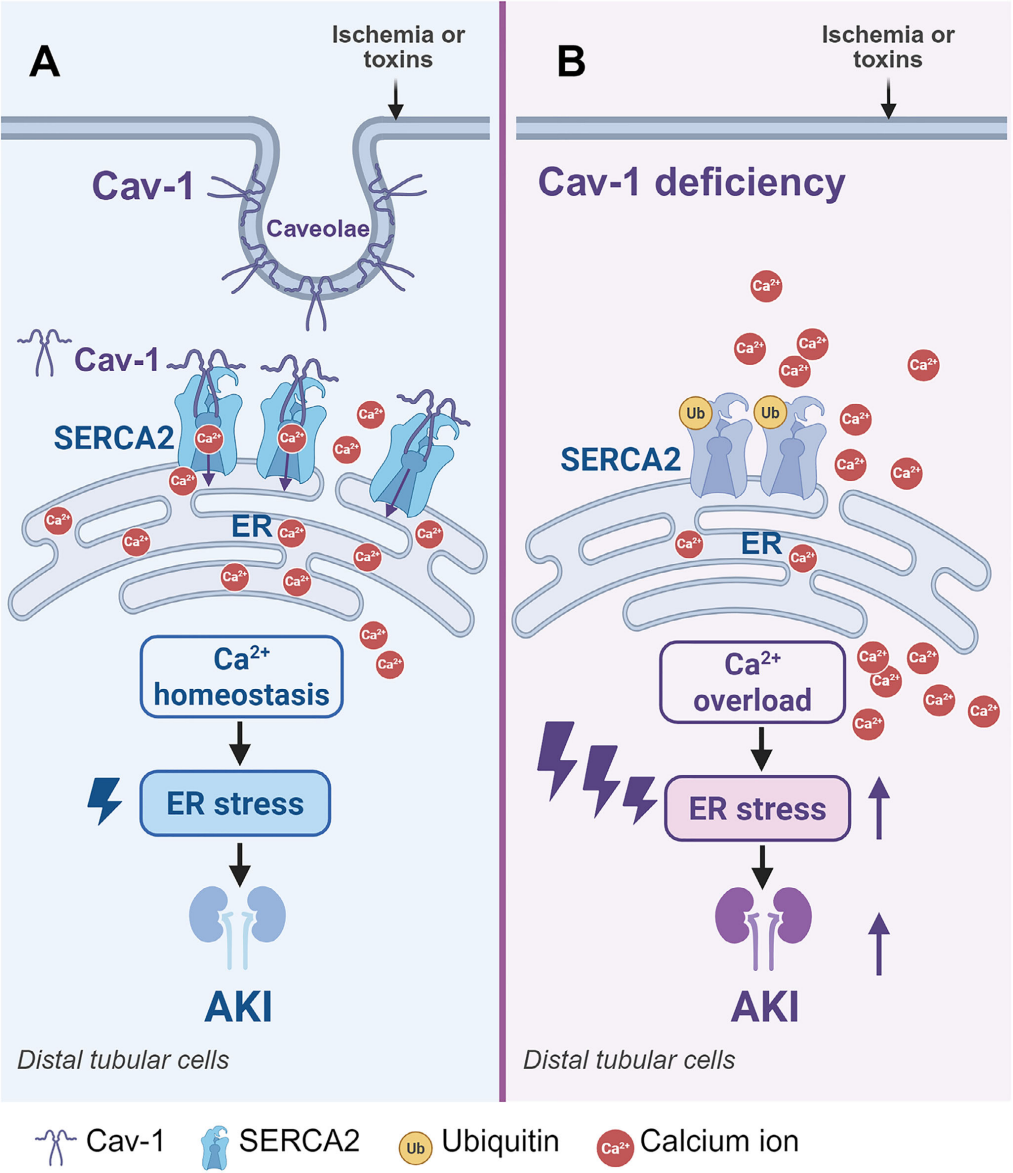
Schematic illustration of the molecular mechanism of Cav‐1 regulating ER stress by stabilizing SERCA2 in AKI. (A) In the presence of Cav‐1, initial kidney injury leads to increasing Cav‐1 expression in distal tubular epithelial cells, increased Cav‐1 directly binds to SERCA2 through its scaffolding domain and stabilizes SERCA2 protein by deubiquitination, thereby regulating Ca^2+^ homeostasis and ER stress in AKI. (B) However, Cav‐1 deficiency in distal TECs significantly accelerates the degradation of SERCA2 protein, thereby increasing Ca^2+^ overload and ER stress, and ultimately exacerbating AKI.

Growing evidence indicates that distal tubules play critical roles in the process of renal injury and repair, in addition to their function to maintain salt‐water and acid‐base homeostasis as recognized traditionally [29, 30]. However, the involvement of distal TECs in AKI remains poorly understood. Here, we demonstrate for the first time that the elevation of Cav‐1 in distal TECs serves as a critical protector against AKI, a finding validated through both global *Cav1* knockout and distal TEC‐specific *Cav1* knockout mouse models. Mechanistically, Cav‐1 interacts with and stabilizes SERCA2 via its scaffolding domain, thereby regulating Ca^2^^+^ homeostasis and inhibiting ER stress (Figure 10). Collectively, our data underscore the significant involvement of distal tubule Cav‐1 in regulating cellular Ca^2^^+^ homeostasis and ER function through the downstream effector SERCA2, and indicate that Cav‐1/SERCA2 may be promising therapeutic targets for the treatment of AKI.

Intracellular Ca^2^^+^ homeostasis is maintained by a tightly regulated system involving Ca^2^^+^ channels, exchangers, and pumps [23]. The ER, which serves as the primary intracellular Ca^2+^ reservoir, playing a critical role in maintaining intracellular Ca^2^^+^ homeostasis [31]. It has been observed that significant intracellular Ca^2^^+^ alterations occur in TECs during I/R‐ and cisplatin‐induced kidney injury, where inhibiting Ca^2^^+^ release from the ER store attenuates I/R‐induced TEC apoptosis [32, 33]. Recent evidence further showed that using a Ca^2+^ chelator or eliminating overloaded Ca^2+^ in damaged TECs can effectively alleviate IRI‐induced AKI in mice, supporting the therapeutic potential of modulating Ca^2^^+^ homeostasis in AKI [34, 35]. Although Ca^2^^+^‐targeting strategies represent an emerging frontier in renal disease, the intricate mechanisms governing Ca^2+^ homeostasis in kidney pathology remain unresolved inquiries. Our investigation provides definitive evidence that Cav‐1 is upregulated in the ER of H/R‐induced RTECs, playing a crucial role in regulating Ca^2+^ homeostasis and ER stress during AKI. Indeed, previous studies in tumor development have identified Cav‐1 as a core component of the Ca^2+^‐dependent apoptotic pathway that regulates critical mitochondrial processes [36, 37], and it can maintain ER function by reducing the activation of UPR, thereby modulating melanoma progression [12]. Our study identifies Cav‐1 as a key molecular link connecting Ca^2+^ homeostasis imbalance and ER stress, offering novel targets for both mechanistic research and treatment of AKI.

An interesting finding in this study is that CSP (a cell‐permeable peptide of Cav‐1) preferentially accumulates in injured renal tissue after systemic delivery and exerts a pronounced protective effect against AKI. This tissue‐specific accumulation in the kidney, rather than in other organs, may be attributed to the unique pathological microenvironment induced by IRI. IRI is a kidney‐specific insult in our model that causes not only tubular damage but also significant endothelial dysfunction and increased vascular permeability, which likely allows circulating CSP to extravasate and specifically accumulate in the injured kidney. Previous study has shown that Cav‐1 deficiency exacerbated choroidal neovascularization in proliferative diabetic retinopathy, while supplementation of CSP inhibited ocular neovascular diseases by inhibiting microglia/macrophage migration through the JNK pathway [13]. In addition, intraperitoneal injection or airway nebulization of CSP suppressed cigarette smoke‐induced lung injury, attenuating mucus hypersecretion, alveolar damage, and significantly improving lung function [38]. Indeed, a Cav‐1‐derived peptide, LTI‐ 03, is undergoing Phase I clinical trial for idiopathic pulmonary fibrosis (IPF), having demonstrated good tolerability in normal volunteers (NCT04233814) and now progressing to a safety trial in IPF patients (NCT05954988). Recently, Komatsu et al. demonstrated that in TGF‐β‐induced lung fibroblasts, exogenous CSP is internalized and accumulates in the ER, where it reduces ER stress and thereby alleviates IPF [39]. This finding aligns with our observation that CSP exerts a renoprotective effect in AKI by improving cellular Ca^2+^ homeostasis and suppressing ER stress in injured distal TECs. Overall, our study provides preclinical evidence that not only expands the therapeutic potential of CSP in AKI but also reveals its novel underlying mechanisms.

Mechanistically, Cav‐1 regulates Ca^2+^ homeostasis and ER function by interacting with SERCA2, which is the key calcium pump that transfers Ca^2+^ from the cytoplasm to the ER lumen [40]. Recent studies in cardiac disease have shown that enhancing the activity and expression of SERCA2 promotes intracellular Ca^2^^+^ reuptake, thereby attenuating myocardial hypertrophy and cardiac dysfunction in a mouse model of heart failure [26, 41]. Moreover, a phase II clinical trial of adeno‐associated virus type 1 (AAV1)/SERCA2 gene therapy in patients with advanced heart failure showed that those receiving the high‐dose AAV1/SERCA2 injection experienced significantly greater improvements in heart function [42]. In this work, we discovered that the expression of SERCA2 in the ER is markedly reduced during AKI, and knockdown of SERCA2 significantly aggravates ER stress and AKI. Furthermore, activation (supplementation with CDN1163) or overexpression of SERCA2 can significantly alleviate intracellular Ca^2^^+^ overload and ER stress in AKI. These results suggest that targeting the activation of the Cav1/SERCA2 axis may represent a novel potential therapeutic approach for treating AKI.

Notably, the absence of Cav‐1 further induces the down‐regulation of SERCA2 expression in AKI, and the therapeutic effect of CSP on ER stress and AKI disappeared in SERCA2 knockdown mice. Previous studies have shown that Cav‐1 serves as a key structural component of mitochondria‐associated ER membrane (MAM), a specialized subdomain of ER, and that *Cav1^−/−^* liver cells displays apparent remodeling of both ER and mitochondria architecture [43]. Additionally, it can form a protein complex with IRE1α in the ER membrane, thereby reducing the level of unfolded protein response stress [12]. Together, these observations raise the possibility that scaffolding of SERCA2 by Cav‐1 localizes SERCA2 within the ER membrane, which serves as a platform for intracellular Ca^2^^+^ transport between the cytoplasm and ER lumen, thereby sustaining Ca^2^^+^ homeostasis and ultimately mitigating ER stress in distal TECs during AKI. However, the specific role of Cav‐1 as a platform participating in the regulation of the complex Ca^2^^+^ homeostasis network in AKI still warrants further exploration in the future.

In conclusion, our study highlights the significant involvement of distal tubular Cav‐1 in the pathogenesis of AKI. Cav‐1 interacts with SERCA2 to maintain Ca^2+^ homeostasis, thereby suppressing intracellular Ca^2+^ overload and ER stress, which ultimately attenuates AKI progression. This knowledge deepens our understanding of the pathological mechanisms of Ca^2+^ dysregulation in AKI and provides potential novel therapeutic directions for improved disease management.

## 4 Experimental Section

### 4.1 Human Kidney Samples

All human renal biopsy samples were obtained from Xiangya Hospital, Central South University, Changsha, China. Consent for this study was obtained from the minimal change disease (MCD) and AKI patients. The diagnosis of AKI was performed according to the Kidney Disease Improving Global Outcomes (KDIGO) guidelines. Clinical protocols were approved by the Ethics Committee of Xiangya Hospital, Central South University (Approval number 2024030537).

### 4.2 Experimental Animals

The Ethics Review Committee for Animal Experimentation of Central South University approved this study. All experimental procedures were in accordance with the National Institute of Health guidelines for the use of experimental animals. *Cav1* knockout (*Cav1^−/−^*) C57BL/6J mice were obtained from the Yuan Lei group from Shanghai Medical College of Fudan University [44]. Male *Cav1^−/−^* and littermate wild‐type (WT) mice were used at the age of 8 to 10 weeks. Cav1‐LoxP mice *(Cav1^fl/fl^*) (C57BL/6JCya‐*Cav1^em1flox^*, Strain S‐CKO‐17614, Cyagen, China) were hybridized with transgenic mice expressing Cre‐recombinase under the cadherin 16 promoter (Cdh16‐Cre) to generate distal tubule‐specific *Cav1* knockout mice (*Cav1^fl/fl^‐Cre*; *Cav1^cko^*). Age‐matched mice without Cre (*Cav1^fl/fl^*) were used as controls. *ATP2a2* (encoded SERCA2)‐LoxP mice *(ATP2a2^fl/fl^*) (GemPharmatech Co, Ltd. Nanjing, China) were hybridized with Cdh16‐Cre mice to generated distal tubule‐specific *ATP2a2* knockdown mice (*ATP2a2^fl/+^‐Cre*). Age‐matched mice without Cre (*ATP2a2^fl/+^*) were used as controls for subsequent experiments. Mouse genotyping was performed using genomic DNA isolated from mouse tails by PCR. All mice were used at the age of 8 to 10 weeks, weighing 22–25 g. The details on the construction of distal tubule‐specific *Cav1* knockout mice and distal tubule‐specific *ATP2a2* gene‐knockdown mice were provided in the Supplementary Methods and Figures S3 and S11.

The IRI‐induced AKI model was established as previously described [45]. Briefly, bilateral renal pedicles were clamped for 30 min using nontraumatic microvascular clips, and then the clips were removed to start reperfusion. Body temperature was maintained at 37.0°C–37.5°C throughout the procedures by using a temperature‐controlled heating device. Mice were sacrificed 12 and 24 h after reperfusion. For prophylactic experiments, CSP was administered via intraperitoneal injection at a dose of 1.5 mg kg^−1^ 12 h prior to renal reperfusion, whereas for therapeutic experiments, the same dose of CSP was administered 1 h post‐reperfusion. For SERCA2 activation, we suspended CDN1163 (Sigma‒Aldrich) in DMSO: TWEEN 80: saline at 10: 10: 80, and then, a dose of 50 mg kg^−1^ body weight was administered via intraperitoneal injection once daily on days 1, 2, or 3 before IRI surgery, as well as 1 h post‐reperfusion.

LPS‐induced AKI mouse model was established through intraperitoneal (i.p.) injection of LPS (Sigma‒Aldrich) at a dose of 10 mg kg^−1^; mice were sacrificed 12 or 24 h later. For prophylactic treatment, CSP was administered 4 h before LPS injection, whereas for therapeutic treatment, CSP was administered 1 h post‐injection.

### 4.3 Peptides Preparation

Peptides corresponding to the putative scaffolding domain of caveolin‐1 (amino acids 82–101; DGIWKASFTTFTVTKYWFYR) or the scrambled control peptide (CP) (WGIDKAFFTTSTVTYKWFRY) were synthesized as a fusion peptide to the C‐terminus of the antennapedia internalization sequence (RQIKIWFQNRRMKWKK), using established methods [19]. These peptides were purified and analyzed by reverse‐phase high‐pressure liquid chromatography and mass spectrometry by Selleck Company. Some peptides were synthesized with a His tag attached to the N‐terminus for tissue distribution studies.

### 4.4 RNA‐seq

RNA‐seq was performed on IRI‐induced littermate WT and *Cav1^−/−^* mice by BerryGenomics (Beijing, China). In brief, RNA was extracted from kidney tissues to assess its integrity and quantity. The transcriptome sequencing library was constructed and sequenced using the Illumina HiSeq 2500 platform after passing quality inspection. Bioinformatics analysis was performed using OmicShare (https://www.omicshare.com/tools).

### 4.5 Gene Expression Omnibus (GEO) Database Extraction

Data from the single‐cell sequencing dataset GSE164647 and RNA‐seq dataset GSE192883 were obtained from the GEO database and utilized for the analysis of *Cav1* expression. Data from a single‐cell RNA sequencing database (http://humphreyslab.com/SingleCell/) was utilized for the analysis of SERCA2 expression. R (version 4.0.4) and RStudio (version 1.2.5033) were used to analyze these data in our study.

### 4.6 Cell Culture

Primary renal tubular epithelial cells (RTECs) were isolated as previously described [46]. Briefly, kidneys were surgically removed from anesthetized WT and *Cav1^−/−^* mice, and the renal cortices were finely chopped and digested with collagenase II (Sigma, 1.0 mg mL^−1^) at 37°C for 10 min. Undigested tissues were removed by filtering through 100‐ and 40‐µm sieves, and the tissue suspensions were diluted and centrifuged at 200 g for 5 min. Then, the supernatants were discarded, and the pure fraction of RTECs was obtained and cultured in DMEM/F12 (Gibco) supplemented with 10% FBS (BioChannel), 1% 10,000 U mL^−1^ penicillin‐streptomycin (Gibco), and incubated at 37°C with 5% CO_2_. To induce the hypoxia/reoxygenation (H/R) in vitro models, cells were exposed to HANKs (Solarbio) in an anaerobic jar (Mitsubishi Gas Chemical Co. Inc) equipped with an AnaeroPack (Mitsubishi Gas Chemical Co. Inc) for 6 h, and for reoxygenation, cells were moved back to normoxic medium with 21% O_2_ and 5% CO_2_, and cells were collected 4 h after reoxygenation for subsequent experiments.

### 4.7 Assessment of Renal Function

Renal functional parameters, including serum creatinine (SCr) and blood urea nitrogen (BUN), were detected using the Creatinine and Urea Assay kit according to the manufacturer's instructions (Nanjing Jiancheng Bioengineering Institute).

### 4.8 Histological Analysis of Renal Tissues

The formalin‐fixed kidneys were embedded in paraffin and cut into 4 µm sections for hematoxylin and eosin (HE) staining. Tubular injury was scored according to the following system: 0 = no injury, 1 = 1% – 25% of area, 2 = 26% – 50% of area, 3 = 51% – 75% of area, and 4 > 75%. Renal tubular injury was defined as necrosis of tubular epithelial cells, loss of brush border, cast formation, dilation, or degeneration.

### 4.9 Immunohistochemical and Immunofluorescence Analysis

Paraffin kidney sections were prepared by a routine procedure. Immunohistochemistry was performed to detect the expression of Cav‐1 (Cell Signaling Technology, Cat#3267) in the kidney according to the established protocol. For immunofluorescence staining of kidney tissues and primary RTECs, samples were permeabilized with 0.1% Triton X‐100 and blocked with 5% BSA in PBS for 1 h at room temperature. Subsequently, samples were immunostained with primary antibodies against Cav‐1 (Proteintech, Cat#66067‐1), SERCA2 (Abcam, Cat#ab150435), Calnexin (Proteintech, Cat#66903‐1), and Calbindin‐D28k (Proteintech, Cat#14479‐1) overnight and then stained with Cy3 or FITC‐conjugated secondary antibody. For the His‐tagged CSP distribution assay, tissue slides were immunostained with primary antibodies against His (Proteintech, Cat#66005‐1) and then stained with a Cy3‐conjugated secondary antibody.

### 4.10 Western Blot Analysis

Kidney tissues or cells were lysed on ice with sodium dodecyl sulfate (SDS) buffer containing a protease inhibitor cocktail (Sigma–Aldrich). The supernatants were collected after centrifugation at 12,000 × rpm at 4°C for 10 min. Protein concentration was measured using a BCA protein assay kit (Thermo Fisher Scientific), and approximately 30 µg protein from each sample was added to validate protein expression. Primary antibodies against Cav‐1 (Cell Signaling Technology, Cat#3267), NGAL (Abcam, Cat#ab318209), GRP78 (Proteintech, Cat#11587‐1), CHOP (Proteintech, Cat#15204‐1), SERCA2 (Abcam, Cat#ab150435), Flag (Proteintech, Cat#66008‐4), GST (Proteintech, Cat#66001‐2), HA (Proteintech, Cat#51064‐2), and β‐actin (Cell Signaling Technology, Cat#3700) were used.

### 4.11 Analysis of mRNA Expression

The expression levels of specific mRNAs were quantified by RT‒qPCR with SYBR Green according to the manufacturer's instructions and were normalized to *β‐actin* mRNA expression. The sequences of the primers are listed in Table S3.

### 4.12 Transmission Electron Microscopy (TEM)

According to the manufacturer's instructions, the renal tissue was cut into 1–2 mm^3^ pieces and fixed in 2.5% glutaraldehyde/0.1 M phosphate buffer at room temperature, and then transferred to 4°C overnight, and examined under an electron microscope.

### 4.13 Confocal Microscopy

We cultured the primary RTECs of WT mice and *Cav1^−/−^* mice in confocal dishes. After dealt with or without H/R, 4% paraformaldehyde was used to fix the cell samples. The images were performed using NIKON ECLIPSE TI inverted laser scanning confocal microscope and photographed by Nikon C2, with the present settings of DAPI (Ex: 405 nm, Em: 417–477 nm), FITC/fluro‐488 (Ex: 488 nm, Em:500–550 nm), TRITC/cy3 (Ex: 561 nm, Em: 570–1000 nm).

### 4.14 Lactic Dehydrogenase (LDH) Release Assay

The release of LDH was detected using the LDH Cytotoxicity Assay Kit (Beyotime Biotechnology, Cat#C0016) according to the manufacturer's instructions. The LDH released into the culture medium of primary RTECs was expressed as a percentage of the total LDH.

### 4.15 Cell Transfection

The mouse and human Cav‐1 overexpression plasmid, human FLAG‐tagged SERCA2 overexpression plasmid, human HA‐tagged ubiquitin plasmid, and four truncated human GST‐tagged Cav‐1 overexpression plasmids were purchased from GenePharma (Shanghai, China). Primary RTECs and HEK293T cells were transfected using Lipofectamine 2000 (Invitrogen) according to the manufacturer's instructions.

### 4.16 Co‐Immunoprecipitation and LC‒MS/MS Analysis

The method of co‐immunoprecipitation (Co‐IP) was carried out according to the instructions (Bimake), and the specific steps were as follows: primary RTECs discarded the medium and washed with 1×PBS for twice; IP lysate buffer (Thermo Fisher Scientific) was added, and the supernatants were collected by centrifugation at 12,000 rpm at 4°C for 10 min. Cav‐1 antibody and normal IgG antibody (Cell Signaling Technology) were added separately and rotated overnight at 4°C. Then, 20 µL Protein A/G magnetic beads were mixed with the antigen‐antibody mixture and rotated at room temperature for 1‒2 h. After washing for 4 times, the remaining protein was extracted by IP lysate buffer for LC‒MS/MS analysis. LC‒MS/MS analysis was performed by Jingjie PTM BioLab (Hangzhou, China) Co. Ltd. Finally, we screened out the substrate proteins that could bind to Cav‐1 according to the score and the mass of detected proteins.

### 4.17 Proximity Ligation Assay (PLA)

Primary RTECs subjected to H/R stimulation were fixed with 4% paraformaldehyde, permeabilized with 0.1% Triton‐X, and blocked. After incubation with anti‐Cav‐1 and anti‐SERCA2 antibodies, the cells were labeled with oligonucleotide‐conjugated secondary antibodies, followed by ligation and amplification using a PLA kit (DUO92002, DUO92004, DUO92008, Duolink Sigma).

### 4.18 Protein Stability Test

For the SERCA2 protein stability test, cycloheximide (CHX, Selleck) or dimethyl sulfoxide (DMSO; Sigma–Aldrich) was added to WT, and *Cav1^−/−^* primary RTECs with 500 µg mL^−1^, and whole cellular lysates were collected at 0, 2, 4, 8 h post‐CHX treatment for western blot analysis using SERCA2 antibody.

### 4.19 MG132 and CQ Treatment

WT and *Cav1^−/−^* primary RTECs were treated with proteasome inhibitor MG132 (10 µm; MedChemExpress, Cat# HY‐13259) or autophagy inhibitor chloroquine (CQ, 10 µm; Selleck, Cat#S6999) dissolved in DMSO for 8 h. Control cells were treated with an equal volume of DMSO alone. The whole cellular lysates were collected for western blot analysis using Cav‐1 and SERCA2 antibodies.

### 4.20 In Vitro Ubiquitination Assays

Human Cav‐1, FLAG‐tagged SERCA2, and HA‐tagged ubiquitin overexpression plasmids were transfected into HEK293T cells for 48 h. All the cells were treated with MG132 at a dose of 10 µm for 6 h before collection. Cells were lysed in IP lysis buffer (denaturing lysis buffer) and heated at 95°C for 10 min. Ubiquitination of SERCA2 was performed by an IP assay using an anti‐Flag antibody (Proteintech, Cat#66008‐4) followed by a western blot analysis with an anti‐HA antibody (Proteintech, Cat#51064‐2).

### 4.21 Intracellular Calcium Ion (Ca^2+^) Detection

WT and *Cav1^−/−^* RTECs were induced by H/R stimulation and added with Fluo‐4 AM working solution (Beyotime Biotechnology) and incubated for 30 min for fluorescence probe loading at 37°C. After washing with PBS twice, the fluorescence intensity of intracellular Ca^2+^ was observed using a fluorescence microscope (Leica).

### 4.22 Statistical Analysis

All data were expressed as the mean ± standard deviation (SD). Statistical analysis was performed with GraphPad Prism software (version 8). A two‐tailed unpaired *t*‐test was used to analyze data between two groups after the determination of data distribution. The ANOVA test was used when more than two groups were present. *p* < 0.05 was considered statistically significant.

## Author Contributions

Yan Zhang: conceptualization, methodology, data curation, formal analysis, funding acquisition, investigation, and writing –original draft. Xin He: data curation, formal analysis, investigation, and writing – original draft. Hao Huang: methodology and formal analysis. Rong Lu: methodology and validation. Xin Lv: methodology and data curation. Shenglan Li: methodology, formal analysis, and supervision. Sijue Zou: methodology and validation. Jiawei Cheng: methodology and validation. Yiwei Xiong: methodology and validation. Zhenghao Deng: formal analysis and supervision. Qiongjing Yuan: formal analysis, funding acquisition, and supervision. Yanyun Xie: methodology and supervision. Ling Huang: formal analysis and supervision. Jiaxi Pu: formal analysis and supervision. Shao Liu: methodology and supervision. Qianbin Li: methodology and supervision. Jie Meng: formal analysis and supervision. Huixiang Yang: formal analysis, funding acquisition, and supervision. Lijian Tao: formal analysis, funding acquisition, supervision, and writing – review. Zhangzhe Peng: conceptualization, formal analysis, funding acquisition, supervision, validation, writing – review and editing.

## Conflicts of Interest

The authors declare no conflicts of interest.

## Supporting information




**Supporting File**: advs75449‐sup‐0001‐SuppMat.docx.

## Data Availability

The data that support the findings of this study are available from the corresponding author upon reasonable request.
