# Supplementary material for: Caveolin‐1 Stabilizes SERCA2 to Counteract Acute Kidney Injury via Suppression of Ca2+‐Dependent Endoplasmic Reticulum Stress in Distal Tubules
Source: Adv Sci (Weinh). 2026 May 8;13(42):e75449. doi: 10.1002/advs.75449 (PMC13335454; doi:10.1002/advs.75449)
Supplement: Supplementary file 1 — Supporting File: advs75449‐sup‐0001‐SuppMat.docx. [file ADVS-13-e75449-s001.docx]

**Supporting Information for**

**Caveolin-1 Stabilizes SERCA2 to Counteract Acute Kidney Injury via Suppression of Ca^2+^-dependent Endoplasmic Reticulum Stress in Distal Tubules**

Yan Zhang^#^, Xin He^#^, Hao Huang, Rong Lu, Xin Lv, Shenglan Li, Sijue Zou, Jiawei Cheng, Yiwei Xiong, Zhenghao Deng, Qiongjing Yuan, Yanyun Xie, Ling Huang, Jiaxi Pu, Shao Liu, Qianbin Li, Jie Meng, Huixiang Yang, Lijian Tao and Zhangzhe Peng*

**
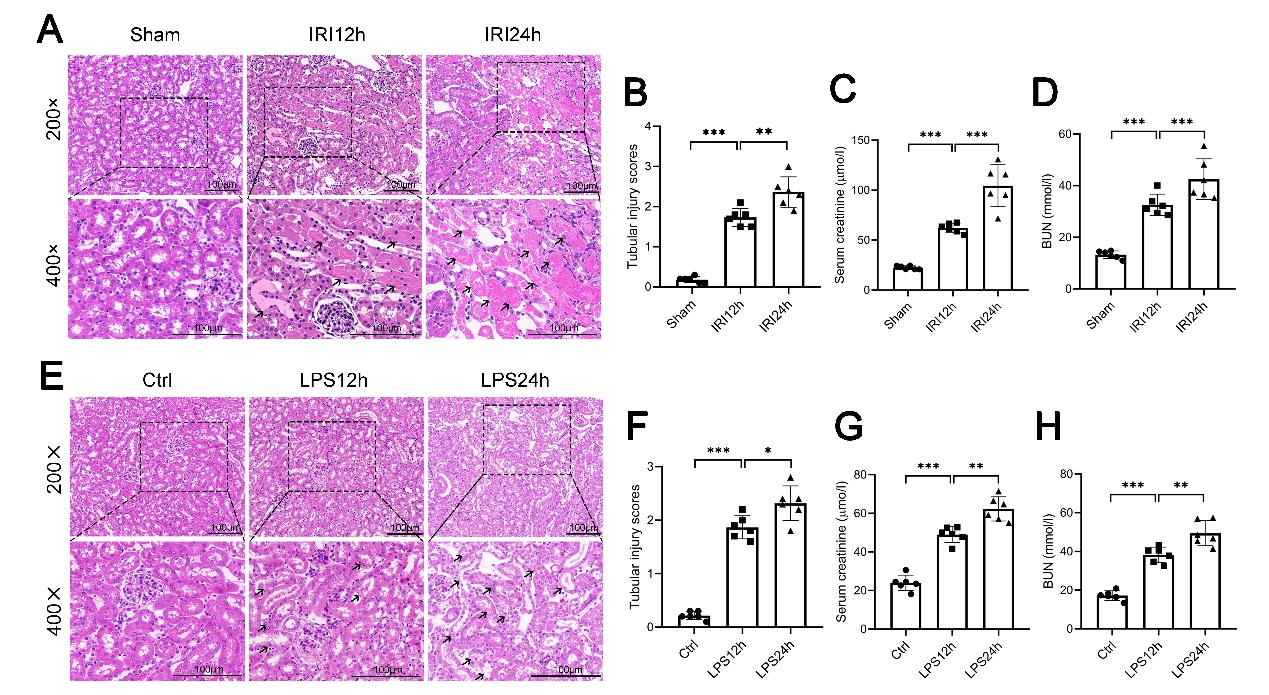
**

**Figure S1. Construction of ischemia/reperfusion injury (IRI) and lipopolysaccharide (LPS) induced acute kidney injury (AKI) models in wide type (WT) mice.** IRI was induced in WT mice and sacrificed 12 or 24 hours after reperfusion. **(A)** Representative images of hematoxylin and eosin (HE) staining of kidney sections from WT mice induced by IRI 12 and 24 hours (n = 6 mice per group). Scale bar = 100 μm. Black arrows indicate injured tubules. (**B)** Tubular injury scores of kidney tissues (n = 6 mice per group). **(C, D)** Serum creatinine (SCr) and blood urea nitrogen (BUN) levels in different groups of WT mice. LPS was injected intraperitoneally (i.p.) in WT mice and sacrificed 12 or 24 hours later. (**E)** Representative images of HE staining of kidney sections from WT mice induced by LPS 12 and 24 hours (n = 6 mice per group). Scale bar = 100 μm. Black arrows indicate injured tubules. (**F)** Tubular injury scores of kidney tissues (n = 6 mice per group). (**G, H)** SCr and BUN levels in different groups of WT mice (n = 6 mice per group). Data are expressed as the mean ± SD. **P* < 0.05, ***P* < 0.01, ****P* < 0.001.

**
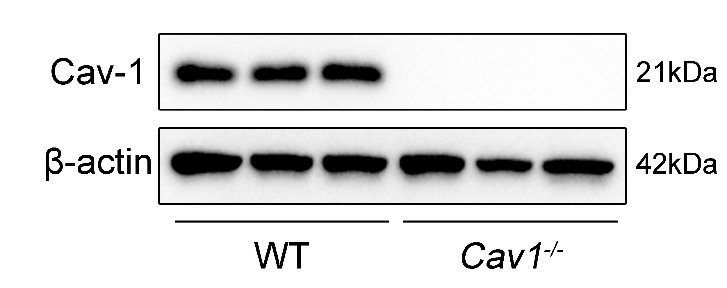
**

**Figure S2. *Cav1* knockout (*Cav1^-/-^*) mice efficiency was measured by western blot.** Western blot analysis of Cav-1 in kidney tissues from WT and *Cav1^-/-^* mice.

**
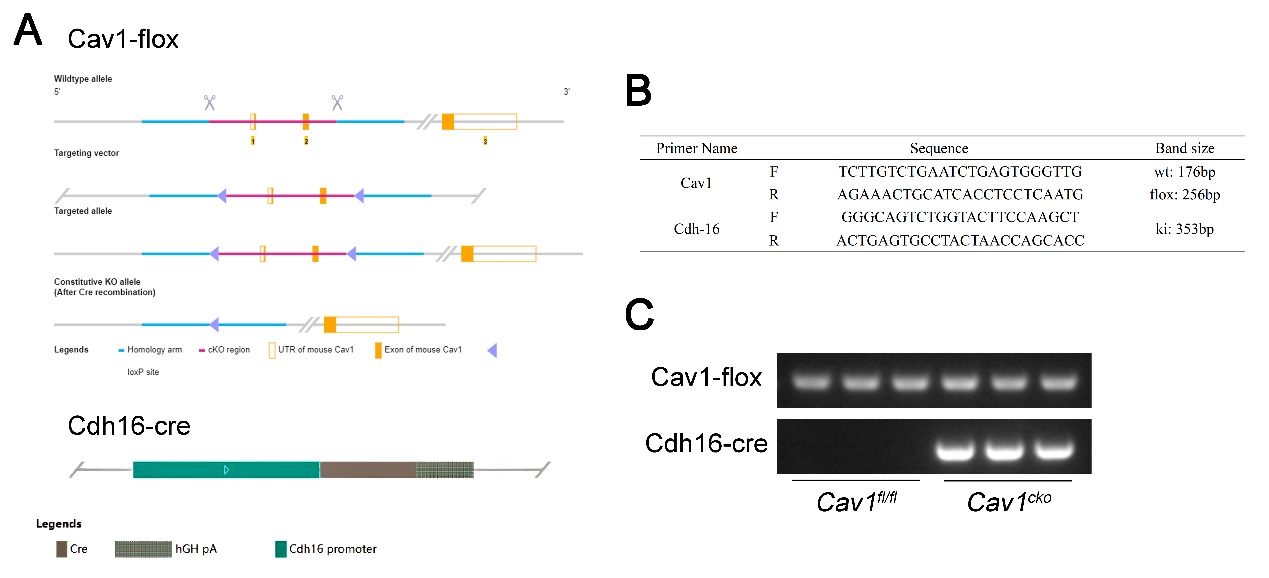
**

**Figure S3. Generation of *Cav1* distal tubular conditional knockout (*Cav1^cko^*) mice. (A)** Schematic of *Cav1^cko^* mice generation. **(B)** The sequences of the primers using for PCR assay. **(C)** Identification of the genotype of *Cav1^cko^* mice and the control mice (*Cav1^fl/fl^*) mice by PCR assay.


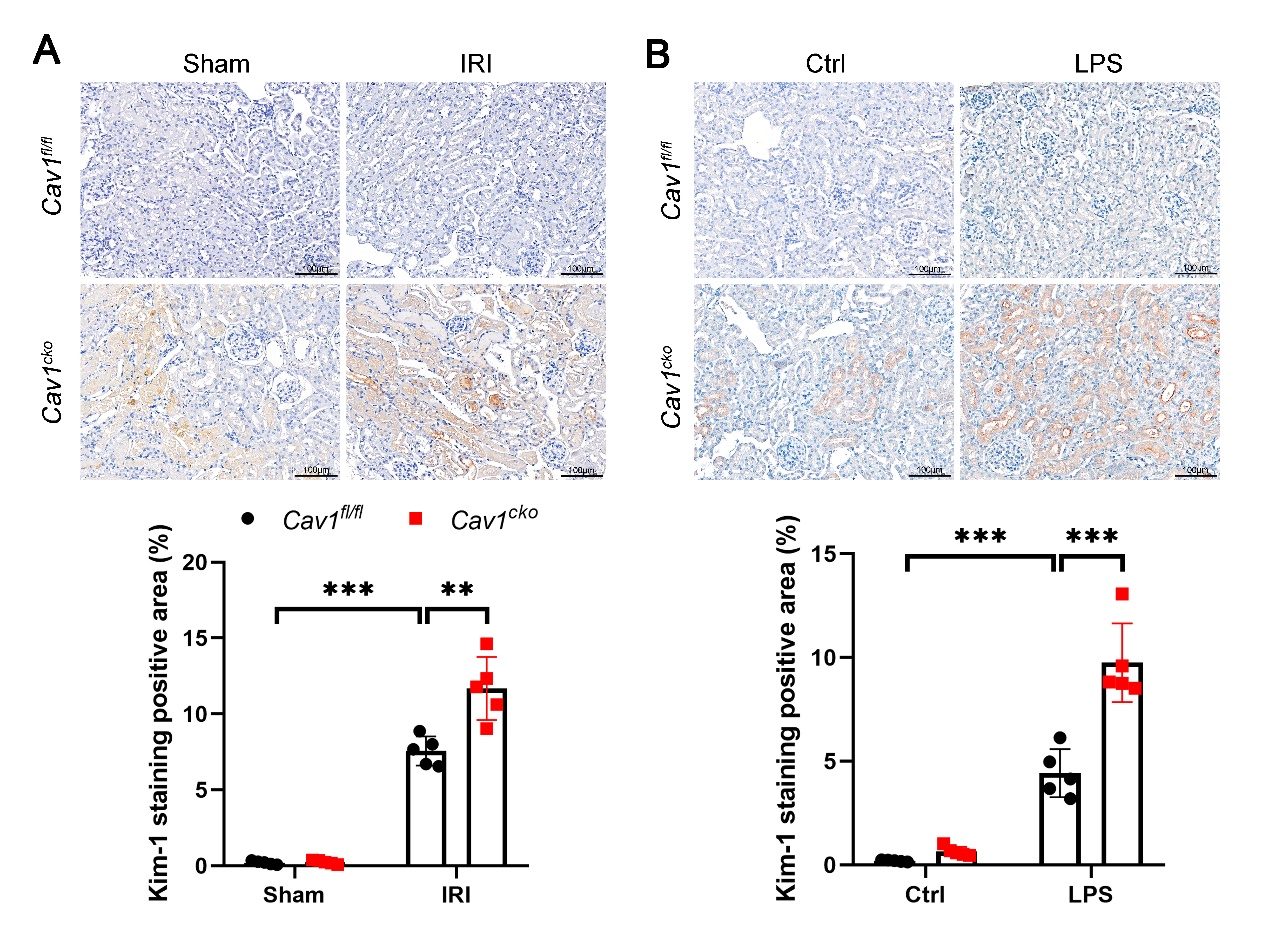


**Figure S4. Kidney injury molecule-1 (Kim-1) is significantly upregulated in kidneys of AKI mice with distal tubule-specific conditional knockout of Cav-1. (A)** Immunohistochemical (IHC) staining of Kim-1 in kidney sections from control (*Cav1^fl/fl^*) and distal tubule epithelial cells (TECs) conditional *Cav-1* knockout (*Cav1^cko^*) mice at 24 hours after IRI (n = 5 mice per group). Scale bar = 100 μm. (B) IHC staining of Kim-1 in kidney sections from *Cav1^fl/fl^* and *Cav1^cko^* mice induced by LPS (n = 5 mice per group). Scale bar = 100 μm. Data are expressed as the mean ± SD. ***P* < 0.01, ****P* < 0.001.

**
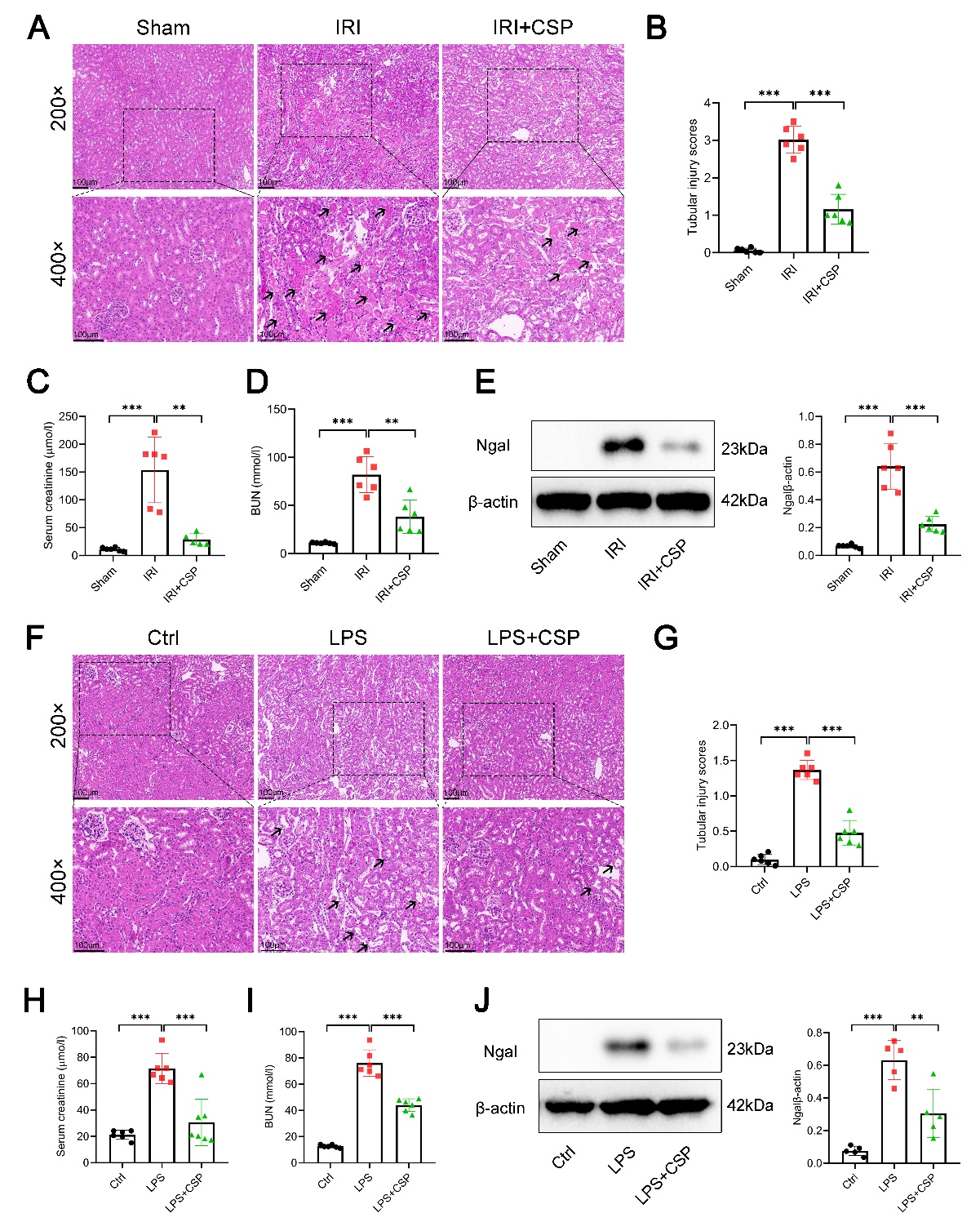
**

**Figure S5. Prophylactic Administration of Cav-1 scaffolding domain peptide (CSP) alleviates IRI and LPS induced AKI in WT mice. (A)** WT mice were injected i.p. with CSP (1.5mg kg ^-1^) or PBS 12 hours before IRI surgery and sacrificed 24 hours after reperfusion. Representative images of HE staining of kidney sections from WT mice induced by IRI with CSP treatment (n = 6 mice per group). Scale bar = 100 μm. Black arrows indicate injured tubules. **(B)** Tubular injury scores of kidney tissues (n = 6 mice per group). **(C, D)** SCr and BUN levels in different groups of WT mice (n = 6 mice per group). **(E)** Western blot analysis and densitometric quantification of Ngal in kidney tissues (n = 6 mice per group). **(F)** WT mice were i.p. injected with CSP or PBS 4 hours before LPS injection and sacrificed 24 hours later. Representative images of HE staining of kidney sections from WT mice induced by LPS with CSP treatment (n = 6 mice per group). Scale bar = 100 μm. Black arrows indicate injured tubules. **(G)** Tubular injury scores of kidney tissues (n = 6 mice per group). **(H, I)** SCr and BUN levels in different groups of WT mice (n = 6 mice per group). **(J)** Western blot analysis and densitometric quantification of Ngal in kidney tissues (n = 5 mice per group). Data are expressed as the mean ± SD. ***P* < 0.01, ****P* < 0.001.


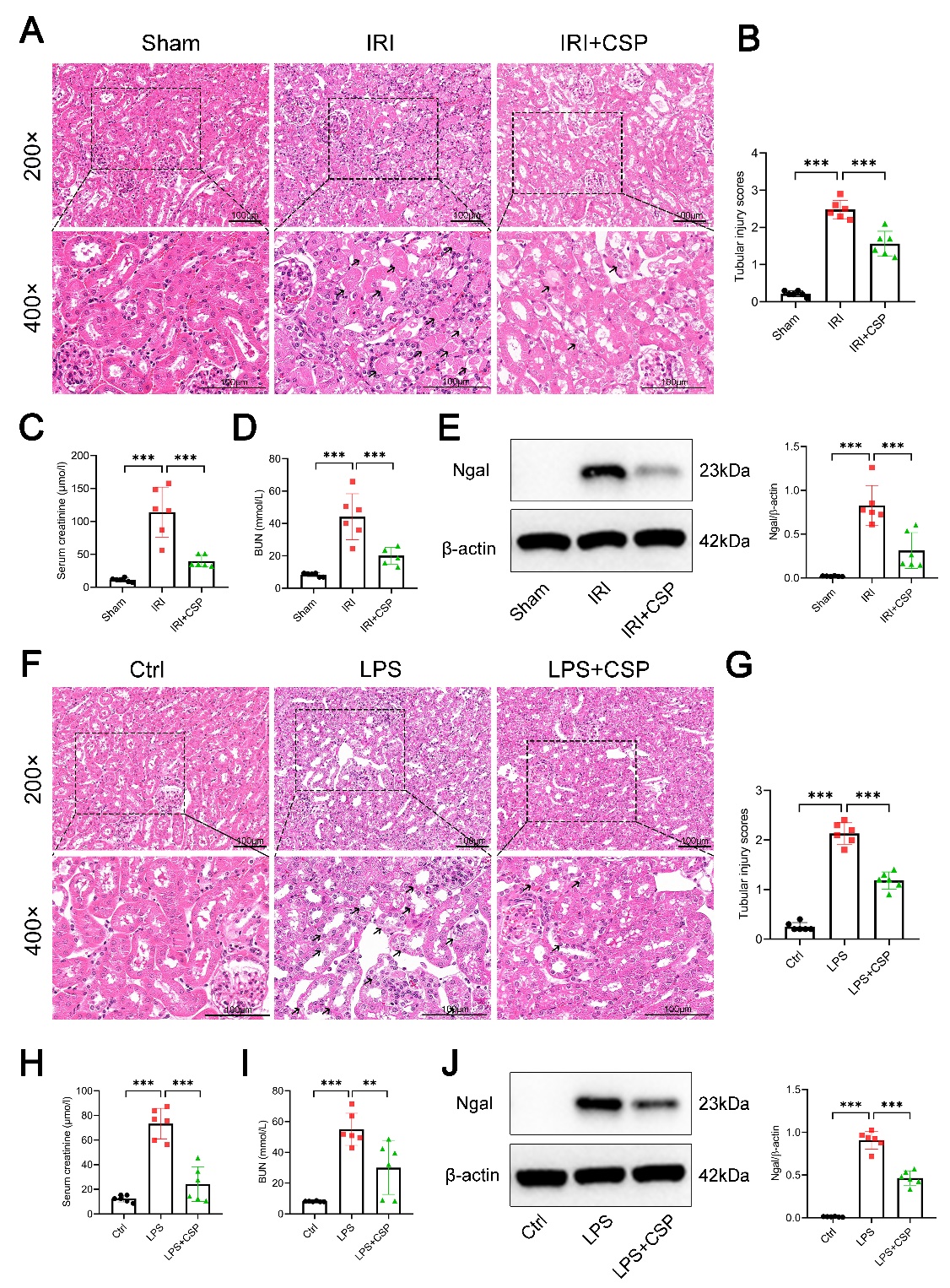


**Figure S6. Therapeutic Administration of CSP alleviates IRI and LPS induced AKI in WT mice. (A)** WT mice were injected i.p. with CSP (1.5mg kg ^-1^) or PBS 1 hour after IRI surgery and sacrificed 24 hours after reperfusion. Representative images of HE staining of kidney sections from WT mice induced by IRI with CSP treatment (n = 6 mice per group). Scale bar = 100 μm. Black arrows indicate injured tubules. **(B)** Tubular injury scores of kidney tissues (n = 6 mice per group). **(C, D)** SCr and BUN levels in different groups of WT mice (n = 6 mice per group). **(E)** Western blot analysis and densitometric quantification of Ngal in kidney tissues (n = 6 mice per group). **(F)** WT mice were i.p. injected with CSP or PBS 1 hour after LPS injection and sacrificed 24 hours later. Representative images of HE staining of kidney sections from WT mice induced by LPS with CSP treatment (n = 6 mice per group). Scale bar = 100 μm. Black arrows indicate injured tubules. **(G)** Tubular injury scores of kidney tissues (n = 6 mice per group). **(H, I)** SCr and BUN levels in different groups of WT mice (n = 6 mice per group). **(J)** Western blot analysis and densitometric quantification of Ngal in kidney tissues (n = 5 mice per group). Data are expressed as the mean ± SD. ***P* < 0.01, ****P* < 0.001.


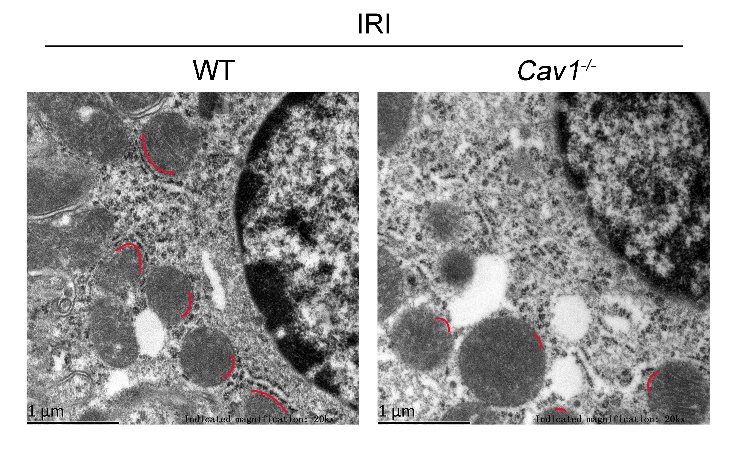


**Figure S7. Cav-1 deficiency reduces endoplasmic reticulum (ER)-mitochondria contact sites in the kidneys of IRI-induced AKI mice.** Typical images of transmission electron microscopy (TEM) of kidney sections from WT and *Cav1^-/-^* mice at 12 hours after IRI. At 20000× magnification, scale bar = 1 μm.


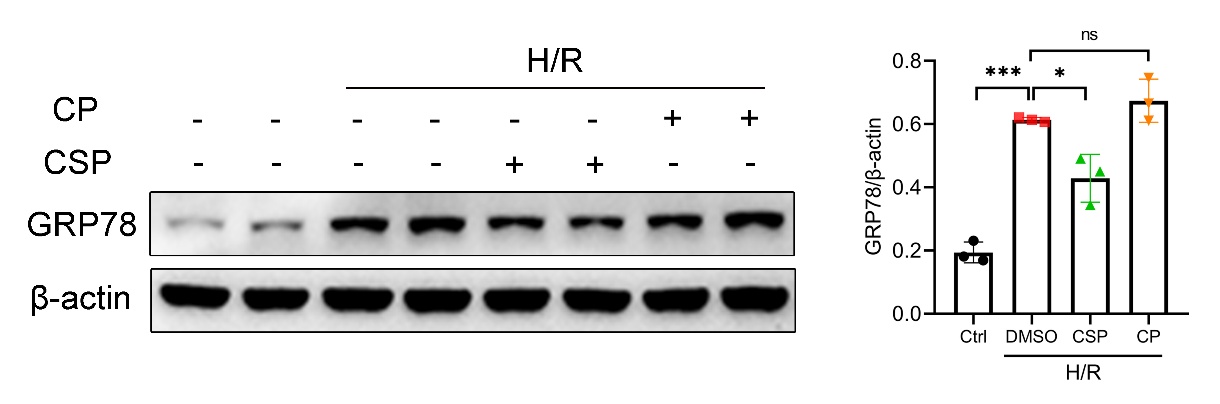


**Figure S8. CSP alleviates hypoxia/reoxygenation (H/R) induced ER stress in *Cav1^-/-^* primary renal tubular epithelial cells (RTECs).** Western blot analysis and densitometric quantification of GRP78 in *Cav1^-/-^* primary RTECs induced by H/R after treatment with CSP (5 μM) or the scrambled control peptide (CP, 5 μM) (n = 3 biologically independent cells). Data are expressed as the mean ± SD. **P* < 0.05, ****P* < 0.001, ns not signiﬁcant.

**
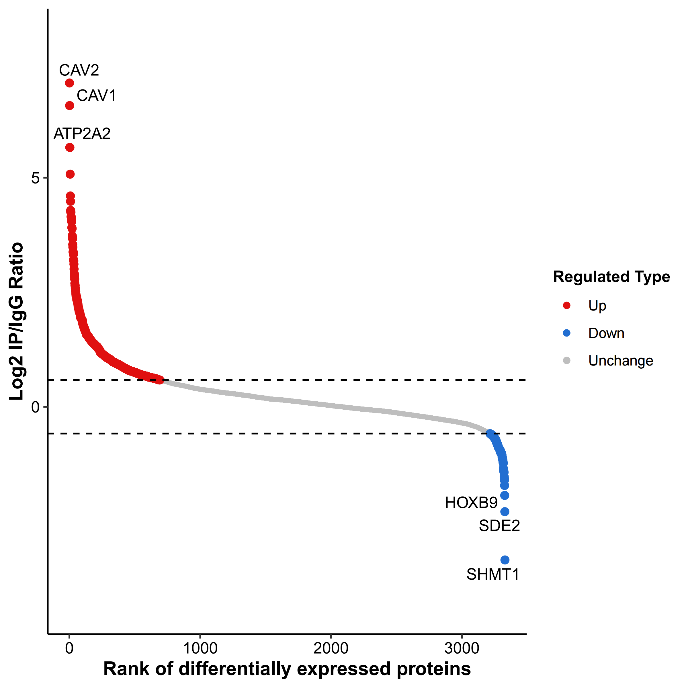
**

**Figure S9. Co-immunoprecipitation (Co-IP) combined with mass spectrometry revealed that sarcoplasmic/endoplasmic reticulum Ca^2+^-ATPase 2 (SERCA2, encoded by *APT2a2*) was the top one candidate interacting protein with Cav-1.**

**
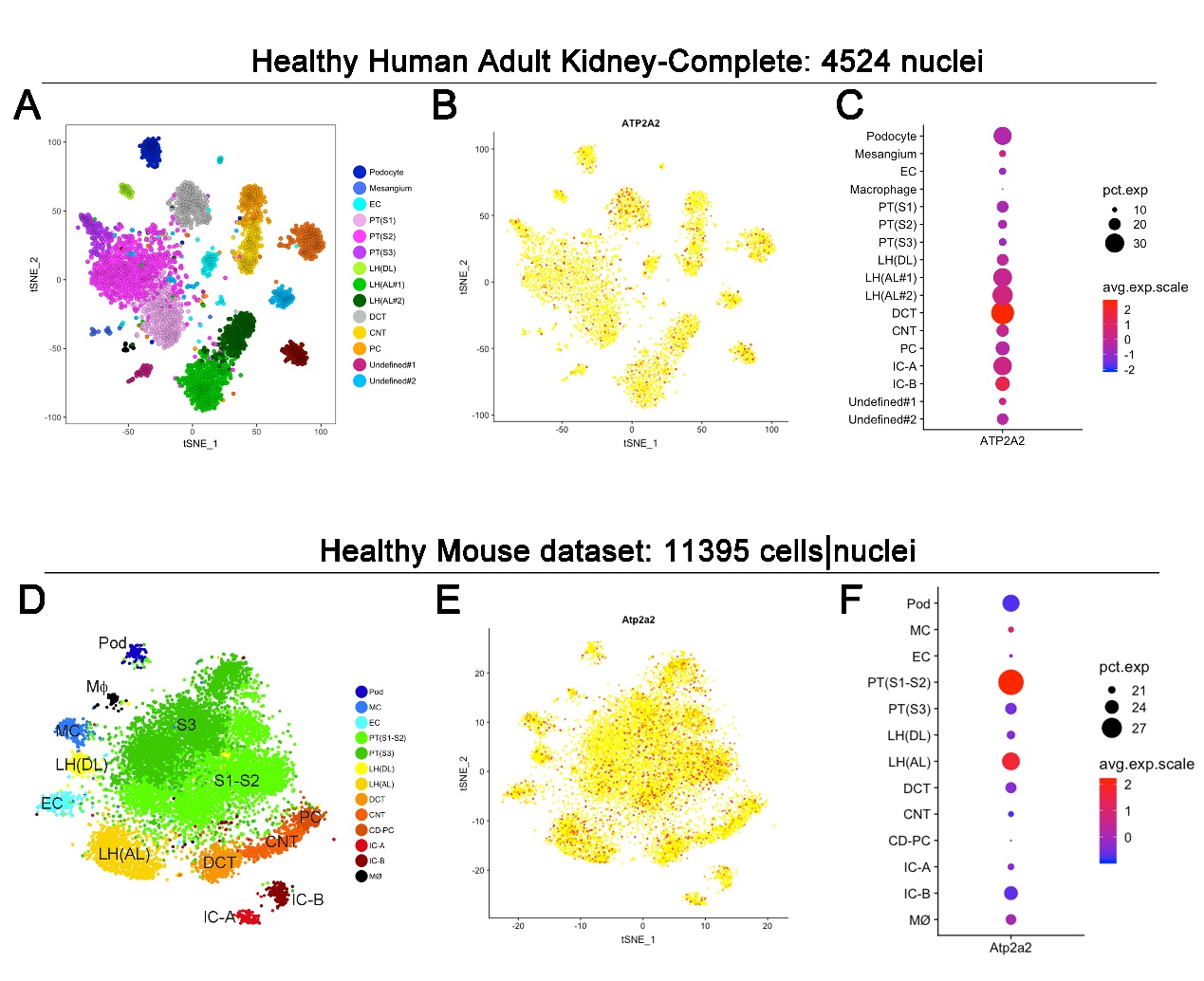
**

**Figure S10. The expression and characteristics of SERCA2 in healthy human adult and healthy mouse kidney single cells.** All open data were from single-cell RNA sequencing database (http://humphreyslab.com/SingleCell/). **(A-C)** Collecting 4524 cells from the healthy adult human kidney, tSNE analysis presented the expression and distribution SERCA2 (encoded by *ATP2a2*) in the kidney cells. **(D-F)** Collecting 11395 cells from the healthy mouse kidney, tSNE analysis presented the expression and distribution SERCA2 (encoded by *ATP2a2*) in the kidney cells. EC, endothelial cells; PT(S1), S1 segment of proximal tubule; PT(S2), S2 segment of proximal tubule; PT(S3), S3 segment of proximal tubule; PT(S1, S2), S1,S2 segment of proximal tubule; LH(DL), loop of Henle descending loop; LH(AL), loop of Henle ascending loop; DCT, distal convoluted tubule; CNT, connecting tubule; Pod, podocyte; MC, mesangial cell; EC, endothelial cell; CD: PC, collecting duct-principal cells; IC-A, intercalated cell type A; IC-B, intercalated cell type B; M.Φ, macrophage.

**
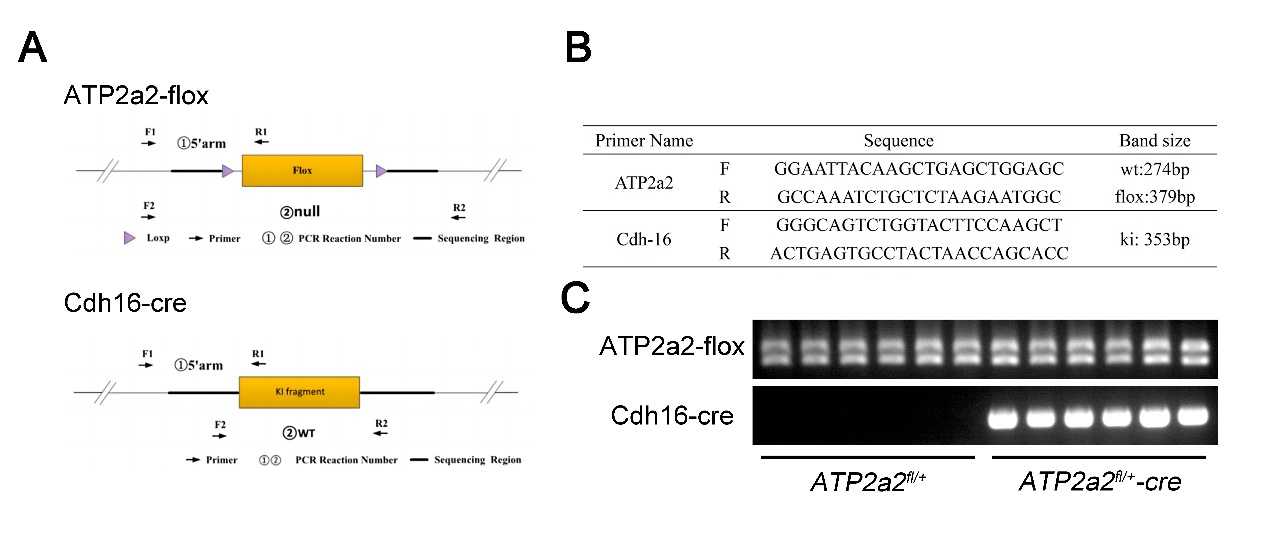
**

**Figure S11. Generation of SERCA2 distal tubular conditional knock-down mice. (A)** Schematic of SERCA2 (encoded by *ATP2a2*) knockdown mice generation. **(B)** The sequences of the primers using for PCR assay. **(C)** Identification of the genotype of SERCA2 distal tubular conditional knock-down mice (*ATP2a2^fl/+^-Cre*) and the control mice (*ATP2a2^fl/+^*) mice by PCR assay.


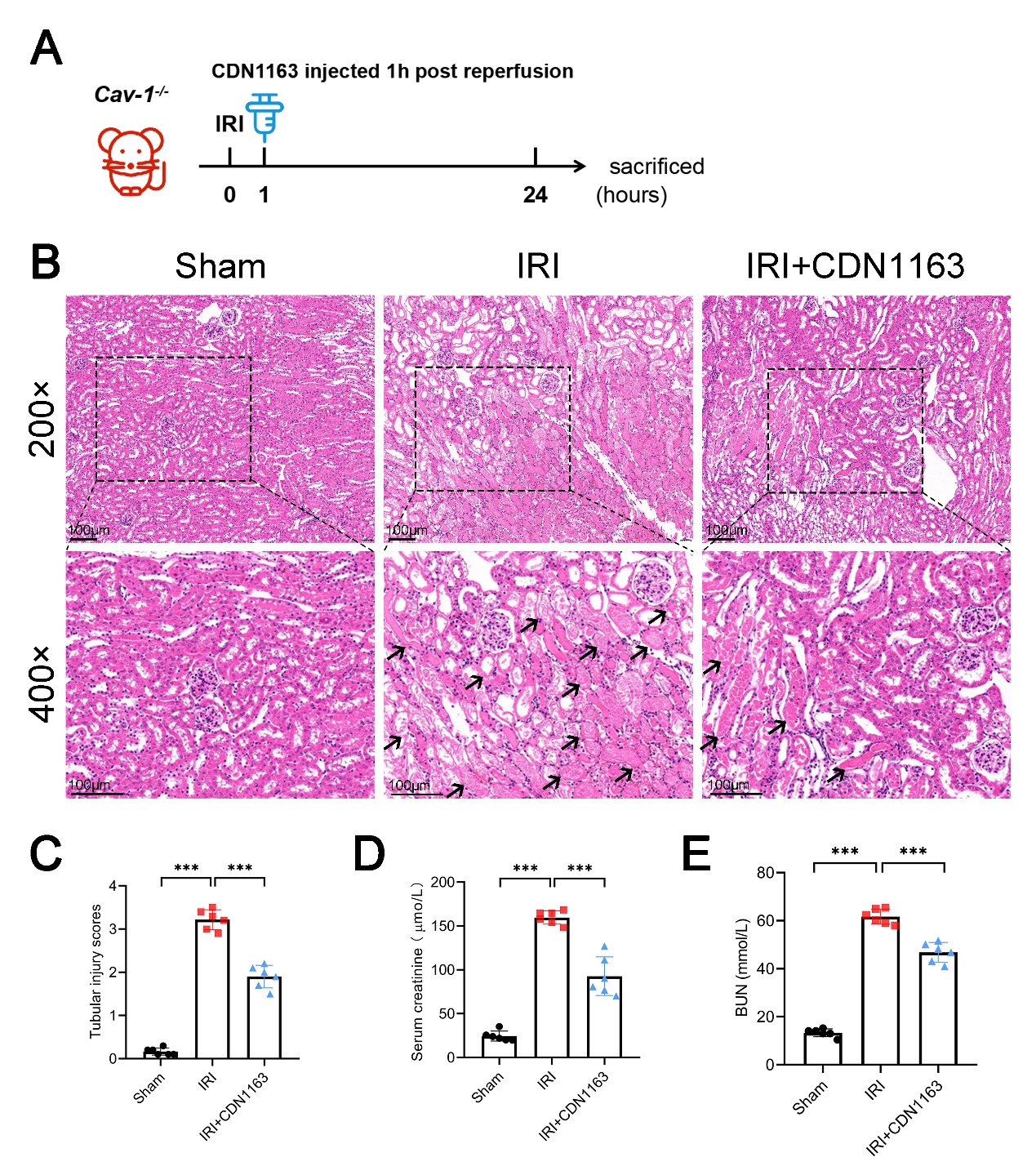


**Figure S12. Therapeutic CDN1163 injection alleviate IRI-induced AKI in *Cav1^-/-^* mice. (A)** The experimental flowchart for the treatment of CDN1163 in *Cav1^-/-^* mice induced by IRI: *Cav1^-/-^* mice were i.p. injected with CDN1163 1 hour post reperfusion and sacrificed 24 hours after reperfusion. **(B)** Representative images of HE staining of kidney sections from *Cav1^-/-^* mice induced by IRI with or without CDN1163 treatment (n = 6 mice per group). Scale bar = 100 μm. Black arrows indicate injured tubules. **(C)** Tubular injury scores of kidney tissues (n = 6 mice per group). **(D, E)** SCr and BUN levels in different groups of *Cav1^-/-^* mice (n = 6 mice per group). Data are expressed as the mean ± SD. ****P* < 0.001.


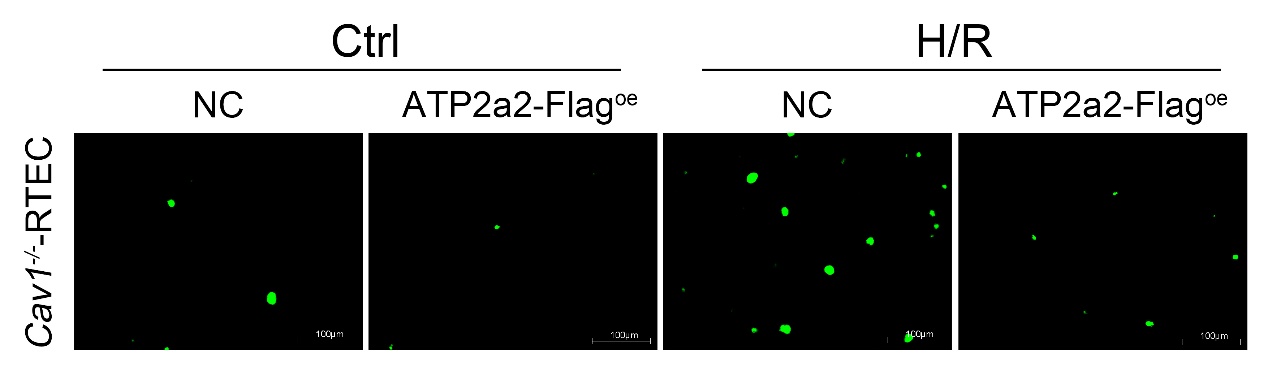


**Figure S13. Overexpression of SERCA2 alleviates H/R induced Ca^2+^ overload in *Cav1^-/-^* primary RTECs.** Representative of Fluo-4AM fluorescence staining of *Cav1^-/-^* primary RTECs treated with negative control (NC) or SERCA2 overexpression (ATP2a2-Flag^oe^) after H/R. Scale bar = 100 μm.

**Table S1 | Characteristics of minimal change disease (MCD) and acute kidney injury (AKI) patients**

| Variable | Normal (n = 10) | AKI (n = 11) |
| --- | --- | --- |
| Gender, n (%) |  |  |
| Male | 8 (80.00%) | 4 (36.36%) |
| Female | 2 (20.00%) | 7 (63.64%) |
| Age (years) | 42.70 ± 9.42 | 49.64 ± 18.38 |
| CBC |  |  |
| RBC (10^12/l) | 4.75 ± 0.29 | 3.45 ± 0.71^***^ |
| WBC (10^9/l) | 5.92 ± 1.82 | 8.26 ± 3.24 |
| HB (g/l) | 148.30 ± 7.90 | 104.80 ± 21.56^***^ |
| PLT (10^9/l) | 209.30 ± 73.49 | 215.00 ± 127.80 |
| Liver function |  |  |
| ALT (U/l) | 31.00 ± 13.15 | 18.35 ± 14.07^*^ |
| AST (U/l) | 28.27 ± 10.86 | 24.69 ± 13.73 |
| Renal function |  |  |
| SCr (μmol/l) | 77.95 ± 12.71 | 231.20 ± 173.00^*^ |
| BUN (mmol/l) | 6.43 ± 3.80 | 12.78 ± 10.66 |
| Uric Acid (μmol/l) | 362.5 ± 121.80 | 334.80 ± 119.00 |

CBC, complete blood count; WBC, white blood cell; HB, hemoglobin; PLT, platelet; ALT, alanine aminotransferase; AST, aspartate aminotransferase; SCr, serum creatinine; BUN, blood urea nitrogen.

^*^*P* < 0.05.

^***^*P* < 0.001.

Values are given as the number or mean ± SD.

**Table S2 | Etiology of acute kidney injury**

| Etiology | N | % |
| --- | --- | --- |
| Infection or sepsis | 4 | 36.36 |
| Drugs | 2 | 18.18 |
| Ischemia | 1 | 9.10 |
| Systemic lupus erythematosus | 1 | 9.10 |
| Multiple myeloma | 1 | 9.10 |
| Rhabdomyolysis | 1 | 9.10 |
| Nephrotic syndrome | 1 | 9.10 |

**Table S3 | Sequences of primers used to amplify the related genes**

| **Name** | **Forward (5’ to 3’)** | **Reverse (5’ to 3’)** |
| --- | --- | --- |
| *Ngal* | GCAGGTGGTACGTTGTGGG | CTCTTGTAGCTCATAGATGGTGC |
| *Chop* | CCACCACACCTGAAAGCAGAA | AGGTGAAAGGCAGGGACTCA |
| *Grp78* | TTCAGCCAATTATCAGCAAACTCT | TTTTCTGATGTATCCTCTTCACCAGT |
| *Atf6* | CTGGGCTCGGTAGTTTGTATC | AGACCTGAATGGCTGCTTAC |
| *Xbp1* | AGCAGCAAGTGGTGGATTTG | GAGTTTTCTCCCGTAAAAGCTGA |
| *Atf4* | AGCCCCACAACATGAC | CCACCTCCAGATAGTCAT |
| *Ire1* | CTGTGGTCAAGATGGACTGG | GAAGCGGGAAGTGAAGTAGC |
| *eif2α* | CAACGTGGCAGCCTTACA | TTTCATGTCATAAAGTTGTAGGTTAGG |
| *Atp2a2* | GGAATTACAAGCTGAGCTGGAGC | GCCAAATCTGCTCTAAGAATGGC |
| *β-actin* | GGCTGTATTCCCCTCCATCG | CCAGTTGGTAACAATGCCATGT |

Ngal, neutrophil gelatinase-associated lipocalin; Chop, CCAAT/enhancer binding protein homologous protein; Grp78, glucose regulated protein 78; Atf6, activating transcription factor 6; Xbp1, x-box binding protein 1; Atf4, activating transcription factor 4; Ire1, inositol-requiring enzyme 1; eif2α, eukaryotic initiation factor 2 alpha; Atp2a2, ATPase, Ca^2+^ transporting, cardiac muscle, slow twitch 2.
